# Ethnic inequalities in positive SARS-CoV-2 tests, infection prognosis, COVID-19 hospitalisations and deaths: analysis of 2 years of a record linked national cohort study in Scotland

**DOI:** 10.1136/jech-2023-220501

**Published:** 2023-07-31

**Authors:** Sarah Amele, Eliud Kibuchi, Ronan McCabe, Anna Pearce, Paul Henery, Kirsten Hainey, Adeniyi Francis Fagbamigbe, Amanj Kurdi, Colin McCowan, Colin R Simpson, Chris Dibben, Duncan Buchanan, Evangelia Demou, Fatima Almaghrabi, Gina Anghelescu, Harry Taylor, Holly Tibble, Igor Rudan, James Nazroo, Laia Bécares, Luke Daines, Patricia Irizar, Sandra Jayacodi, Serena Pattaro, Aziz Sheikh, Srinivasa Vittal Katikireddi

**Affiliations:** 1 MRC/CSO Social and Public Health Science Unit, School of Health and Wellbeing, University of Glasgow, Glasgow, UK; 2 Public Health Scotland, Glasgow Office, Glasgow, UK; 3 Institute of Applied Health Sciences, University of Aberdeen, Aberdeen, UK; 4 Department of Epidemiology and Medical Statistics, Faculty of Public Health, College of Medicine, University of Ibadan, Ibadan, Oyo, Nigeria; 5 Strathclyde Institute of Pharmacy & Biomedical Sciences (SIPBS), Faculty of Science, University of Strathclyde, Glasgow, UK; 6 Department of Pharmacology,College of Pharmacy, Hawler Medical University, Erbil, Kurdistan, Iraq; 7 School of Medicine, University of St Andrews, St Andrews, Fife, UK; 8 Usher Institute of Population Health Sciences and Informatics, The University of Edinburgh, Edinburgh, UK; 9 School of Health, Wellington Faculty of Health, Victoria University of Wellington, Wellington, New Zealand; 10 Centre for Research on Environment, Society and Health, School of GeoSciences, Institute of Geography, University of Edinburgh, Edinburgh, UK; 11 Research Data Scotland, Edinburgh, UK; 12 Scottish Centre for Administrative Data Research (SCADR), University of Glasgow, Glasgow, UK; 13 Department of Global Health and Social Medicine, King's College London, London, UK; 14 Department of Sociology, School of Social Sciences, The University of Manchester, Manchester, UK; 15 Patient and Public Involvement (PPI) Representative, Non affiliated, Glasgow, UK

**Keywords:** COVID-19, ethnic groups, health inequalities, epidemiology, public health

## Abstract

**Background:**

This study aims to estimate ethnic inequalities in risk for positive SARS-CoV-2 tests, COVID-19 hospitalisations and deaths over time in Scotland.

**Methods:**

We conducted a population-based cohort study where the 2011 Scottish Census was linked to health records. We included all individuals ≥ 16 years living in Scotland on 1 March 2020. The study period was from 1 March 2020 to 17 April 2022. Self-reported ethnic group was taken from the census and Cox proportional hazard models estimated HRs for positive SARS-CoV-2 tests, hospitalisations and deaths, adjusted for age, sex and health board. We also conducted separate analyses for each of the four waves of COVID-19 to assess changes in risk over time.

**Findings:**

Of the 4 358 339 individuals analysed, 1 093 234 positive SARS-CoV-2 tests, 37 437 hospitalisations and 14 158 deaths occurred. The risk of COVID-19 hospitalisation or death among ethnic minority groups was often higher for White Gypsy/Traveller (HR 2.21, 95% CI (1.61 to 3.06)) and Pakistani 2.09 (1.90 to 2.29) groups compared with the white Scottish group. The risk of COVID-19 hospitalisation or death following confirmed positive SARS-CoV-2 test was particularly higher for White Gypsy/Traveller 2.55 (1.81–3.58), Pakistani 1.75 (1.59–1.73) and African 1.61 (1.28–2.03) individuals relative to white Scottish individuals. However, the risk of COVID-19-related death following hospitalisation did not differ. The risk of COVID-19 outcomes for ethnic minority groups was higher in the first three waves compared with the fourth wave.

**Interpretation:**

Most ethnic minority groups were at increased risk of adverse COVID-19 outcomes in Scotland, especially White Gypsy/Traveller and Pakistani groups. Ethnic inequalities persisted following community infection but not following hospitalisation, suggesting differences in hospital treatment did not substantially contribute to ethnic inequalities.

**WHAT IS ALREADY KNOWN ON THIS TOPIC**

- Ethnic minority groups are disproportionately at a higher risk of COVID-19 outcomes in the UK.
- Most studies have focused on aggregated categories of ethnicity, which has often masked important heterogeneity in COVID-19 outcomes among ethnic minorities.
- Most studies on ethnic inequalities in COVID-19 outcomes have focused on differences in incidence, with less evidence on differences following confirmed positive SARS-CoV-2 test and COVID-19-related hospitalisation.

**WHAT THIS STUDY ADDS**

- We investigated ethnic inequalities in positive SARS-CoV-2 tests, severe COVID-19 (hospitalisation or death) and COVID-19 hospitalisation or death following a confirmed positive SARS-CoV-2 test and death in Scotland.
- We found heterogeneity in risks of COVID-19 outcomes within subcategories of aggregated ethnic groups.
- Ethnic minority groups experienced higher risks of COVID-19 hospitalisation or death, which persisted after restricting analyses to those with a confirmed positive SARS-CoV-2 test.

**HOW THIS STUDY MIGHT AFFECT RESEARCH, PRACTICE OR POLICY**

- This finding is important for policy-making, as it shows the existence of differences in risk within aggregated ethnic groups and the need for tailored interventions.

## Introduction

Data from several countries have highlighted the disproportionate impact of SARS-CoV-2 infection and COVID-19 among some ethnic minority groups.^1–4^ Reasons for the variation observed between ethnic minority groups include differences in exposure to SARS-CoV-2, differential vulnerability to COVID-19 disease due to pre-existent health and socioeconomic disadvantage, differential outcomes to the racialised public health responses to control the pandemic and differential exposure to vaccines, monoclonal antibodies and antiviral treatments.^3^ In the UK, previous work has shown COVID-19 has particularly affected Pakistani, Bangladeshi and black African and Caribbean populations disproportionately.^5–7^

Understanding at which stage ethnic inequalities in COVID-19 outcomes arise can help inform policy within this pandemic and in the future.^3^ The majority of the previous literature on ethnic inequalities in COVID-19 outcomes has focused on differences in incidence,^2 8^ whereas the literature on differences in prognosis (ie, outcomes after confirmed positive SARS-CoV-2 tests and COVID-19-related hospitalisation)^9^ is limited. Moreover, a clear understanding of changes in ethnic inequalities across COVID-19 waves can potentially provide insights into how policies may have exacerbated or mitigated inequalities over time and provide insight to help drive improvements in the delivery of care.

COVID-19 studies have mainly focused on broad categories of ethnicity due to the poor quality of coding within many health datasets, small sample sizes of ethnic minority groups, and undertheorised approaches to ethnic inequalities across these groups.^10 11^ This may often lead to an underestimation of the magnitude of ethnic inequalities in health, since broad categories often mask important heterogeneity.^12^ For example, some white ethnic minority groups who have poorer health profiles than the white majority population are often ignored.^12^ This necessitates the use of more specific ethnic subcategories, which allows for a more granular exploration of ethnic inequalities compared with broad categories. This can be achieved by linking health datasets to self-reported ethnicity from the census, which will improve epidemiological surveillance.

This study aimed to investigate ethnic inequalities in positive SARS-CoV-2 tests and severe COVID-19 outcomes (hospitalisations or deaths) in Scotland. Also, COVID-19 hospitalisations or deaths following a confirmed positive SARS-CoV-2 test, and deaths following COVID-19 hospitalisation, were explored. We additionally evaluated changes in ethnic inequalities over time across the four waves of increased transmission of COVID-19.^13^

## Methods

### Study design and population

Data from the Early Pandemic Evaluation and Enhanced Surveillance of COVID-19 (EAVE II) study^14^ were linked to the 2011 Scottish Census,^15^ which was the source for the study’s primary exposure (ethnicity). We included all individuals who were alive, aged ≥16 years and residents of Scotland on 1 March 2020.^14 15^ The study period was from 1 March 2020 (date of first COVID-19 case in Scotland) to 17 April 2022.

The EAVE-II study includes approximately 99% of the Scottish population and includes primary care, testing, vaccination, hospitalisation and mortality data.^14^ These data are linked together using the Community Health Index (CHI), a unique numeric identifier used by National Health Service Scotland within health records, which records registrations and deregistration with primary and secondary care services.^15^ SARS-CoV-2 testing data were taken from the Electronic Communication of Surveillance in Scotland, morbidity and mortality data from Scottish Morbidity Record and National Records of Scotland datasets, respectively.

The 2011 Scottish Census took place on 27 March 2011 and achieved an overall response rate of 94% among the estimated population of 5.3 million individuals. Ethnic classification was based on 16 groups, with ethnic minority groups constituting 4% of the Scottish population. The approximate linkage rate of 2011 census to the EAVE-II study datasets was 94%.

### Outcomes

The primary outcome was COVID-19-related hospitalisation or death (severe COVID-19). We also explored COVID-19 hospitalisation and death as separate outcomes, in addition to RT-PCR-confirmed positive SARS-CoV-2 test. It is important to note that a ‘positive SARS-CoV-2 test’ represents having tested positive for SARS-CoV-2, which requires individuals to have been first tested. A COVID-19-related hospitalisation was defined based on the International Classification of Diseases—10 code (U07.1 and U07.2) as the reason for admission (any position), or presence of an RT-PCR confirmed positive test for SARS-CoV-2 in the 28 days prior to admission.^16^ COVID-19-related death was defined as either a death where U07.1 and U07.2 were recorded as the primary or secondary causes of death or any death where the individual had a positive RT-PCR test for SARS-CoV-2 infection in the 28 days prior to death. We also undertook a sensitivity analysis where a positive SARS-CoV-2 test was listed as the only primary cause of COVID-19-related hospitalisation or death. In this case, we did not include COVID-19-related hospitalisations or death that preceded a confirmed positive SARS-CoV-2 test.

To understand pathways to ethnic inequalities, we also calculated differences in the risk of COVID-19-related hospitalisation or death following confirmed positive SARS-CoV-2 test and COVID-19-related deaths following confirmed COVID-19-related hospitalisation.

### Ethnicity

Self-reported ethnicity was defined based on 5 aggregated categories (and 16 disaggregated subcategories) in the 2011 Scottish Census classification:

- White: white Scottish, white other British, white Irish, White Gypsy/Traveller, white Polish, other white.
- Mixed or multiple ethnic groups (hereafter mixed).
- Asian: Pakistani, Pakistani Scottish or Pakistani British (Pakistani), Indian, Indian Scottish or Indian British (Indian), Bangladeshi, Bangladeshi Scottish or Bangladeshi British (Bangladeshi), Chinese, Chinese Scottish or Chinese British (Chinese), other Asian.
- Black or African: African, African Scottish or African British (African), Caribbean, Caribbean Scottish, Caribbean British (Caribbean).
- Other ethnic groups (hereafter other): Arab, Arab Scottish or Arab British (Arab) and other disaggregated.

For the analyses of aggregated groups, ethnicity was determined using the 2011 Scottish Census as the default choice and supplemented by a Public Health Scotland (PHS) ethnicity look-up variable, which was used to impute missing values. The PHS ethnicity look-up variable was generated using the most recent ethnicity data from primary care, hospitalisations, secondary care, outpatients, laboratory and unscheduled care.^14^ When carrying out disaggregated analyses, we considered only those individuals with non-missing ethnicity values in the 2011 census due to the likely misclassification of more specific ethnic groups within health datasets.^10^ Individuals with missing disaggregated ethnic data were those who responded to the 2011 census but did not provide their ethnicity. Ethnic differences in baseline characteristics were described based on age and sex.

### Statistical analysis

Baseline characteristics (as of 1 March 2020) were presented using summary statistics per ethnic group. Differences in COVID-19 outcomes: confirmed infection, hospitalisation, death and both hospitalisation and death were also described by ethnicity.

We used Cox proportional models to estimate HRs with 95% CI for the risk of each of the COVID-19 outcomes by ethnicity. Analyses were conducted for the full study period (1 March 2020 to 17 April 2022). Secondary analyses to assess differences across the four waves of increased transmission of COVID-19: wave 1 (1 March 2020 to 31 July 2020), wave 2 (1 August 2020 to 30 April 2021), wave 3 (1 May 2021 to 17 December 2021) and wave 4 (18 December 2021 to 17 April 2022) were also undertaken.^13^

All analyses were adjusted for age (5-year bands), sex and health board (a regional authority in Scotland with responsibility for the delivery of health services). Since ethnicity is a complex and multidimensional social construct, we did not adjust for variables such as socioeconomic position or clinical risk factors because they are mediating variables for ethnic inequalities and their inclusion may result in misleading interpretations due to overadjustment.^3^ We did a sensitivity analysis excluding the health board, as it was unclear if it could be a mediator or not. Individuals were followed from the beginning of the study period until their first event of interest or the end of the study period (17 April 2022), whichever occurred first. Observations and events for ethnic groups containing small numbers (≤10) were removed as per data disclosure agreements. All analyses were conducted in the Scottish National Safe Haven using R statistical software (V.4.0.2), with R codes available on https://github.com/Kibuchi-eliud/Ethnic_inequalities_in_Scotland_COVID19.git in GitHub.

## Results

**Figure 1:**
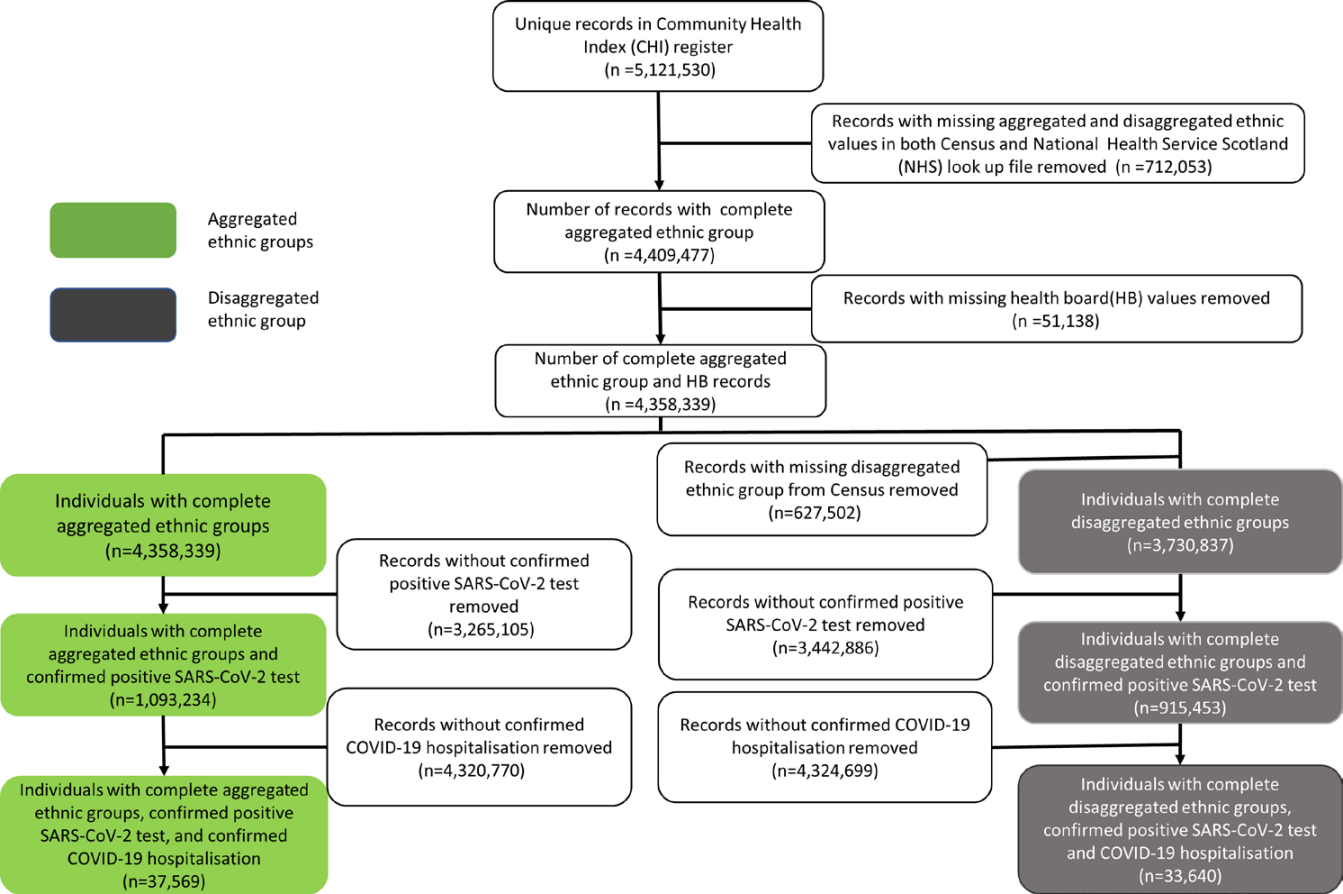
Study population flow chart.

A total of 5 121 530 individuals were registered in the Scottish CHI on 1 March 2020 (figure 1) and were therefore considered the eligible population. We excluded 712 053 (13.90%) individuals with unavailable ethnicity values in either census or PHS look-up file. Also, 51 138 (1.00%) individuals with health board missing values were excluded. Of the total eligible population, 4 358 339 (85.10%) made up the final cohort to study aggregated groups; 1 093 234 (21.35%) had confirmed positive SARS-CoV-2 tests and 37 569 (0.73%) had a confirmed hospitalisation. The final cohort with complete disaggregated subcategories consisted of 3 730 837 (72.85%) individuals; of which 915 453 (17.87%) and 33 640 (0.66%) had confirmed positive SARS-CoV-2 test and hospitalisation, respectively.

Most of the study population (95.0%) were white, followed by Asian (3.1%), black or African (0.8%) and mixed (0.5%) (table 1). There were slightly more females (51.8%) than males (48.2%), and the mean age was highest in the white group (50.0 years, SD=19.3) and lowest in the mixed group (34.5 years, SD=14.1). The characteristics of disaggregated groups were suppressed as per disclosure policy.

**Table 1:** Baseline characteristics by aggregated ethnic group as at 1 March 2020

Ethnic group		White, n (%)	Mixed, n (%)	Asian, n (%)	Black or African, n (%)	Other group, n (%)	Total, n (%)
Total		4 142 573	22 379	135 729	32 933	24 725	4 358 339
Sex	Male	1 995 806 (48.2)	10 445 (46.7)	65 716 (48.4)	16 565 (50.3)	12 685 (51.3)	2 101 217 (48.2)
	Female	2 146 767 (51.8)	11 934 (53.3)	70 013 (51.6)	16 368 (49.7)	12 040 (48.7)	2 257 122 (51.8)
Age in years	Mean	50.0	34.5	39.2	38.40	37.7	49.4
	SD	19.3	14.1	15.4	13.10	13.8	19.3
COVID-19 outcomes	Hospitalisations or deaths	41 247 (1.0)	85 (0.4)	913 (0.7)	183 (0.6)	130 (0.5)	42 558 (1.0)
	Hospitalisations	34 983 (0.8)	81 (0.4)	850 (0.6)	181 (0.5)	*	
	Deaths	13 393 (0.3)	11 (0.1)	187 (0.1)	11 (0.1)	*	
	Positive SARS-CoV-2 test	1 013 428 (24.5)	6047 (27.0)	33 744 (24.9)	9229 (28.0)	6097 (24.7)	1 068 545 (24.5)
Confirmed positive SARS-CoV-2 test	Hospitalisations or deaths	37 839 (0.9)	81 (0.4)	864 (0.6)	176 (0.5)	123 (0.5)	39 083 (0.9)
	Hospitalisations	33 171 (0.8)	78 (0.3)	815 (0.6)	174 (0.5)	*	
	Deaths	11 522 (0.3)	10 (0.1)	168 (0.1)	11 (0.1)	*	
Confirmed COVID-19 hospitalisations	Deaths	7129 (0.2)	124 (0.6)	*	*	*	

*Small numbers not allowable for disclosure release.

**Figure 2:**
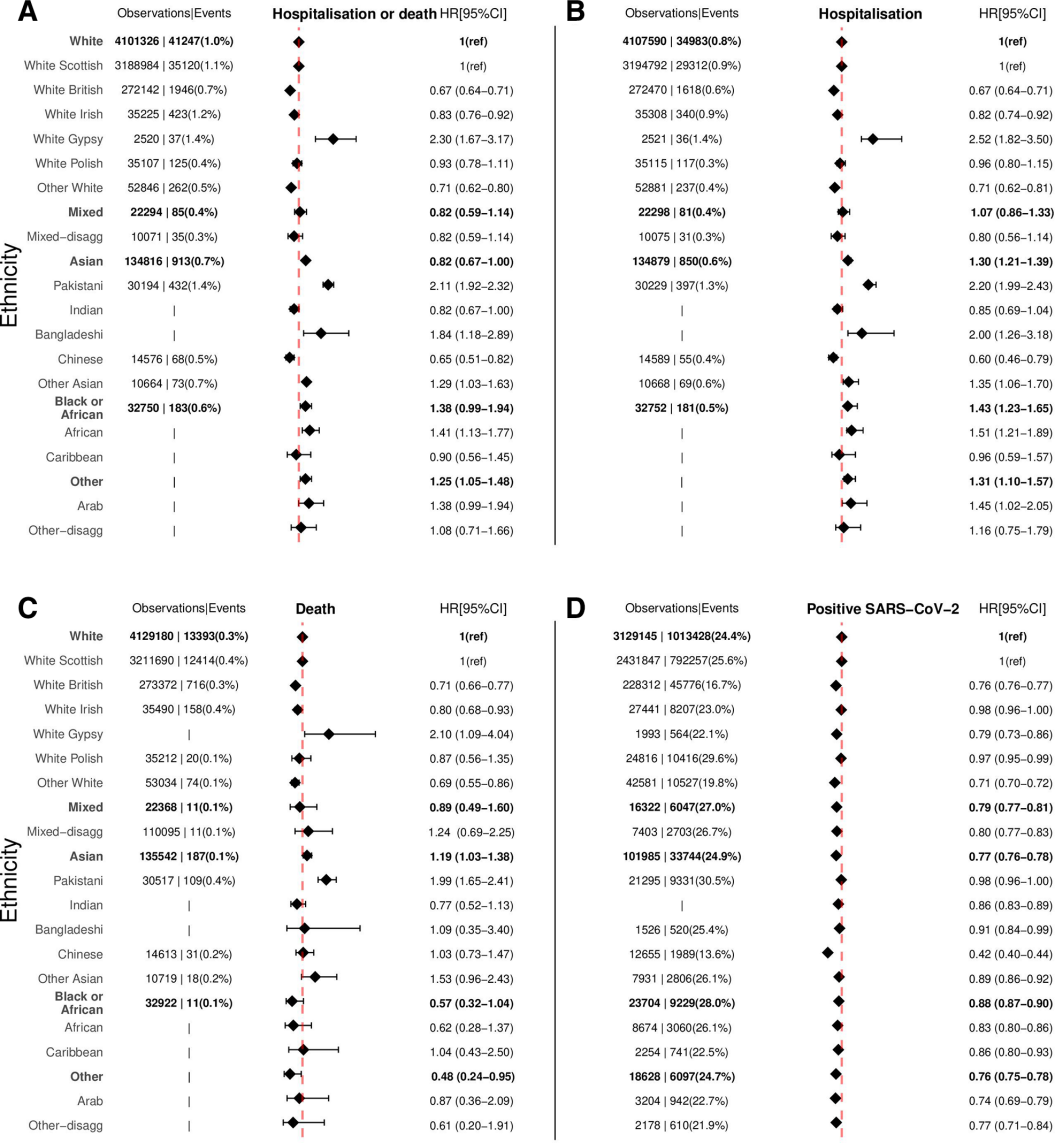
Ethnic differences in the risk of COVID-19-related outcomes for: (A) hospitalisation or death, (B) hospitalisation, (C) death and (D) positive SARS-CoV-2 test. Models adjusted for age, sex and health board. Data are observations and events. Observations and Events for ethnic groups containing small numbers (≤10) were removed as per data disclosure agreements and indicated by only |. White ethnic group is the reference category for comparison of ethnicity in the aggregated categories, and white Scottish is the reference category for comparison of ethnicity in the disaggregated subcategories.

Ethnic minority groups had higher risks of COVID-19 hospitalisation or death after adjusting for age, sex and health board, especially White Gypsy/Traveller adjusted HR 2.30 (95% CI: 1.67 to 3.17), Pakistani 2.11 (1.92 to 2.32), Bangladeshi 1.84 (1.18 to 2.89), other Asian 1.29 (1.03 to 1.63) and African 1.41 (1.13 to 1.77), relative to white Scottish (figure 2, online supplemental tables S1 and S2). In contrast, the Chinese group had a lower risk 0.65 (0.51–0.82). The pattern of risks for hospitalisations only was similar to the combined risk of hospitalisations or deaths across the disaggregated groups. For COVID-19-related death, the risks were elevated for Pakistani 2.20 (1.79–2.70) and other Asian 1.80 (1.10–2.94) groups relative to white Scottish. The risks of confirmed positive SARS-CoV-2 test were lower across all disaggregated minority groups compared with the white Scottish.

10.1136/jech-2023-220501.supp1 Supplementary data

**Figure 3:**
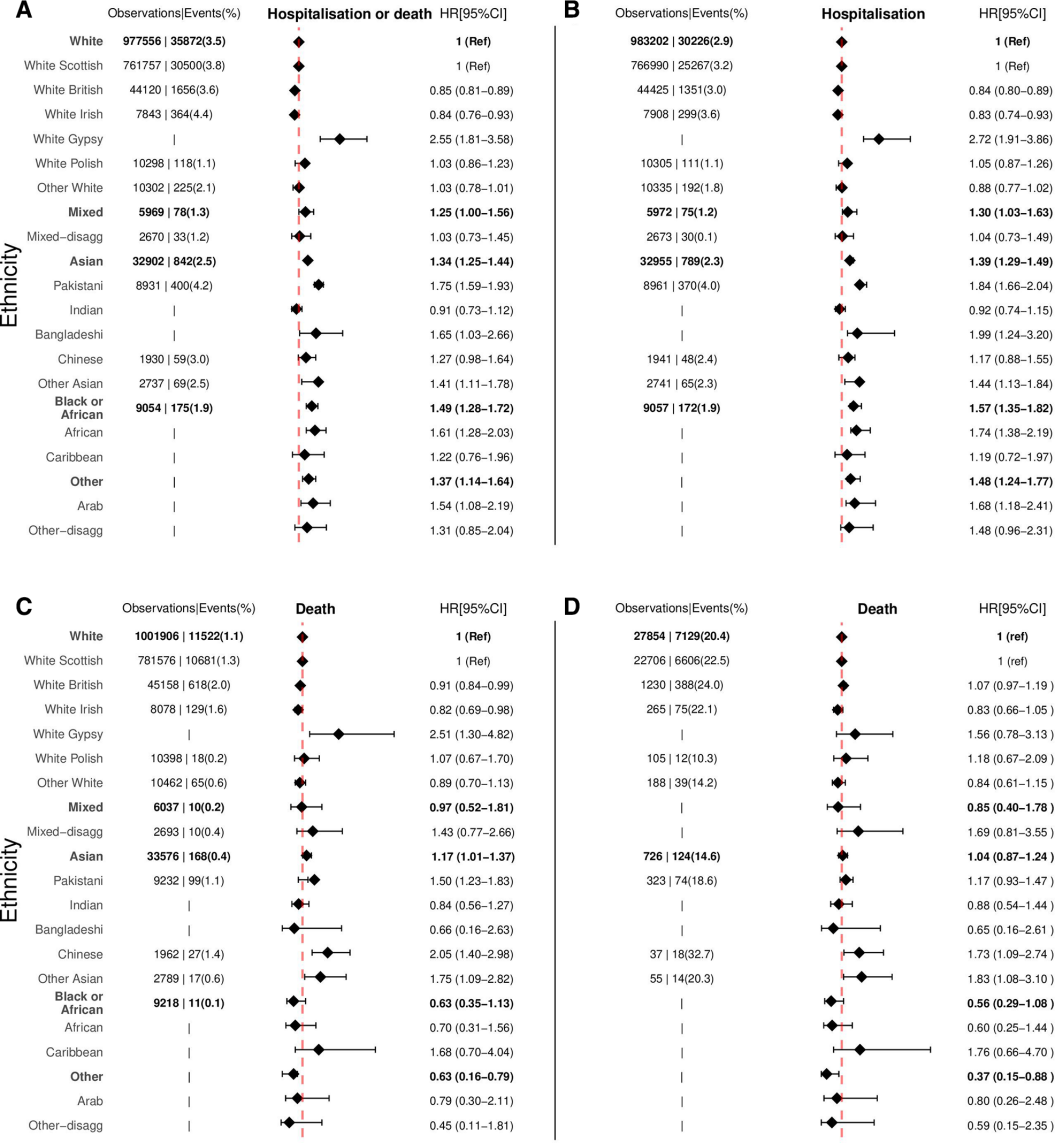
Ethnic differences in the risk of COVID-19-related outcomes for: (A) hospitalisation or death following confirmed infection, (B) hospitalisation following confirmed positive SARS-CoV-2 test, (C) death following confirmed positive SARS-CoV-2 test and (D) death following COVID-19 hospitalisation. Models adjusted for age, sex and health board. Data are observations and events. Observations and Events for ethnic groups containing small numbers (≤10) were removed as per data disclosure agreements and indicated by only |. White ethnic group is the reference category for comparison of ethnicity in the aggregated categories, and white Scottish is the reference category for comparison of ethnicity in the disaggregated subcategories.

For the aggregated groups, Asian: 1.26 (1.18–1.46), black or African: 1.35 (1.17–1.56) and other 1.26 (1.06–1.50) ethnic groups had higher risks of COVID-19 hospitalisation or death relative to the white group. The pattern for COVID-19 hospitalisation only was similar to hospitalisations or deaths for the aggregated groups. The risk of COVID-19-related deaths was higher in the Asian group 1.19 (1.03–1.38) compared with the white group, while risk did not significantly differ in the black or African 0.57 (0.32–1.04) and other 0.48 (0.24–0.95) groups relative to white group. The risk of positive SARS-CoV-2 test was lower across all minority groups compared with the white group. Sensitivity analysis results where the primary cause of hospitalisation or death was solely COVID-19 (online supplemental figure S1, online supplemental tables S3 and S4) were slightly elevated when compared with those obtained when COVID-19-related admission was from any position. However, the overall risk pattern across ethnic groups was similar.

Relative to the white group, ethnic minority groups were more likely to be hospitalised or die following a positive SARS-CoV-2 test (figure 3, online supplemental tables S5 and S6). The difference in risks for hospitalisations or deaths following positive SARS-CoV-2 test was slightly higher compared with those obtained for the unrestricted population. The pattern for the risk of COVID-19-related deaths was also similar to that of the unrestricted population.

Across disaggregated groups, the risks of hospitalisation or death following positive SARS-CoV-2 test for White Gypsy/Traveller 2.55 (1.81–3.58), Pakistani 1.75 (1.59–1.93), Bangladeshi 1.65 (1.03–2.66), other Asian 1.41 (1.11–1.78), African 1.61 (1.28–2.03) and Arab 1.54 (1.08–2.19) groups were higher relative to white Scottish. These risks were slightly higher compared with those for incidence among the whole population, especially among the Arab group. The pattern of risks for hospitalisations for disaggregated subcategories were similar to those for hospitalisations or deaths following positive SARS-CoV-2 test. The risks of COVID-19-related deaths following confirmed positive SARS-CoV-2 test were higher for White Gypsy/Traveller and Pakistani groups relative to white Scottish, and slightly higher compared with those obtained from the whole population. The risk for death for Chinese 2.05 (1.40–2.98) and other Asian 1.75 (1.08–2.81) groups was greater than those seen for incidence among the whole population.

The risk of death following COVID-19 hospitalisation did not differ for Asian and black or African groups when compared with the white group. However, the other group had a lower risk 0.37 (0.15–0.88). Across disaggregated, ethnic minority groups had similar risks to the white Scottish, except among the Chinese 1.73 (1.12–2.82) and other Asian 1.83 (1.08–3.10) groups where risks were slightly higher. Where the primary cause of hospitalisation or death was solely COVID-19 (online supplemental figure S2 and tables S7 and S8), the estimates were similar to those obtained when COVID-19 was included alongside other conditions.

**Figure 4:**
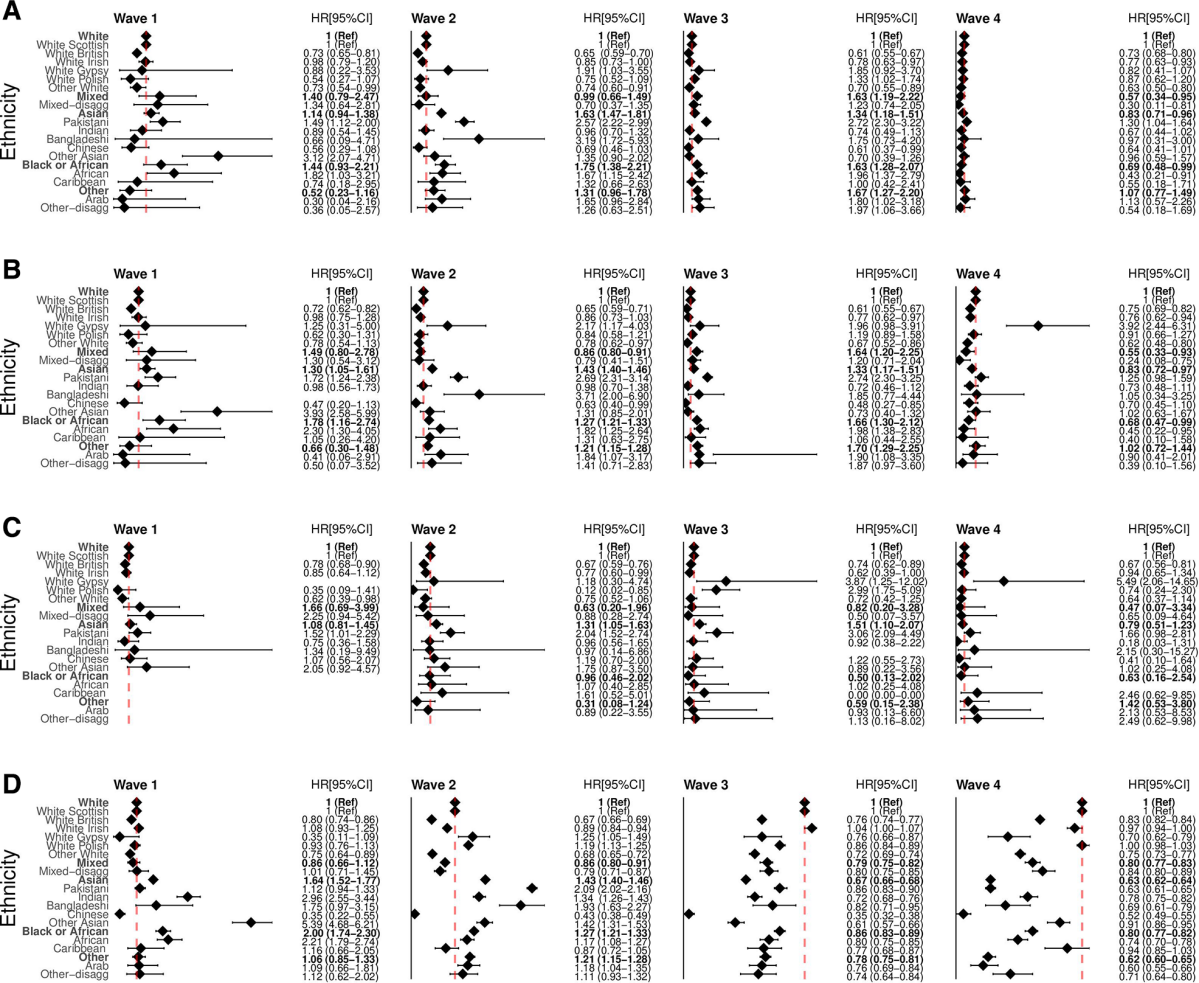
Ethnic differences in the risk of COVID-19-related outcomes for: (A) hospitalisation or death, (B) hospitalisation, (C) death and (D) positive SARS-CoV-2 test across four waves. Models adjusted for age, sex and health board. Bold estimates are for aggregated ethic groups with white group as the reference category. The non-bold estimates are for disaggregated ethnic groups with white Scottish as the reference category. The bold and non-bold HR estimates are not comparable, since they are based on different reference categories. Wave 1 is from 1 March 2020 to 31 July 2020; wave 2 from 1 August 2020 to 30 April 2021; wave 3 from 1 May 2021 to 17 December 2021; and wave 4 from 18 December 2021 to 17 April 2022.

Ethnic minority groups experienced a higher risk of hospitalisations or deaths at the start of the pandemic (ie, waves 1–3), which was no longer present by wave 4, except among the White Gypsy/Traveller group (figure 4, online supplemental tables S9 and S10). The risk of death among white Polish was higher in wave 3 compared with white Scottish 2.99 (1.75–5.09) despite not being different over the whole period. For positive SARS-CoV-2 test, the Indian, other Asian and African groups started out with a higher risk compared with the white Scottish group, but ended up with a lower risk in wave 4. This is in contrast with the lower risks of confirmed infections observed over the whole period.

## Discussion

In this whole population study of ethnic inequalities in COVID-19 outcomes in Scotland, we have found heterogeneity in risks of severe COVID-19 outcomes across ethnic minority groups with White Gypsy/Traveller and Pakistani groups having particularly higher risks compared with white Scottish groups. Moreover, for aggregated groups, the risk of severe COVID-19 outcomes was higher in Asian and black or African groups compared with the white group. The higher risks of COVID-19 hospitalisation or deaths experienced by ethnic minority groups in the whole population persisted when analyses were restricted to those with confirmed positive SARS-CoV-2 test. However, no ethnic differences in the risk of COVID-19-related deaths were found following COVID-19 hospitalisation. Comparisons across the four waves of the pandemic revealed a higher risk of COVID-19 outcomes for ethnic minority groups in the first three waves, which lessened over time.

Our findings show that ethnic minority groups have a greater risk of severe COVID-19-related outcomes are consistent with previous findings conducted in England using linked census data.^7 17 18^ This has been associated with high rates of existing chronic conditions and worse socioeconomic position experienced by ethnic minority groups,^19^ with racism as an underlying contributor to both socioeconomic inequalities and chronic conditions.^20^ The identification that the White Gypsy/Traveller and Pakistani groups have a particularly higher risk of severe COVID-19 outcomes confirms the possibility that important heterogeneity in risks across subcategories of ethnic minority groups is hidden when aggregated groups are used in analysis.^5^ This finding is important because a recent systematic review identified only a few studies exploring ethnic inequalities in COVID-19 outcomes across ethnic subcategories, with white ethnic minority groups rarely studied.

Most studies have assessed inequalities in incidence, but fewer have looked at prognosis, which might help indicate how equitable healthcare is.^21^ Our findings are consistent with the broader evidence showing the existence of ethnic inequalities in COVID-19-related hospitalisation and deaths.^4 21^ This suggests that ethnic minority groups experience underdiagnosis and are likely to face barriers to testing, which relates to their lower infection risk observed in this study.^17 22^ However, it is unclear if differential exposure and vulnerability to infection/disease accounts for all ethnic inequalities or if differential consequences of disease do also contribute.

Our findings that ethnic minority groups have no increased risk of death following hospitalisation are consistent with results of two systematic reviews.^4 21^ This suggests that secondary care provided for COVID-19 patients in Scotland is equitable across ethnic groups as has also been shown in England.^9 21^ However, Chinese and other Asian groups were more likely to die following hospitalisation, despite having a lower risk of hospitalisation compared with white Scottish individuals. This may be caused either by higher morbidity or severity of symptoms at the point of admission.

It was clear that ethnic minority groups had an increased risk of COVID-19 outcomes at the beginning of the pandemic (ie, waves 1–3). However, this risk reduced by wave 4^23 24^ which may reflect either acquired herd immunity due to vaccination efforts and infection^25^ or decreasing severity of new variants.^25–27^

### Strengths and limitations

First, we used self-reported ethnicity from the 2011 Scottish National Census linked with timely COVID-19 primary care, testing, vaccination, hospitalisation and mortality data. This reduces the risk of ethnic groups being misclassified, especially when analysing disaggregated ethnic groups. Second, this study is population based with nationwide coverage, which reduces the risk of selection bias of the individuals included in the study. Third, we also explored ethnic inequalities in COVID-19 outcomes for subpopulations following confirmed positive SARS-CoV-2 test and following COVID-19 hospitalisation. This is important for policy-making as it shows at which stage to implement policies aimed at reducing ethnic inequalities for COVID-19 and future pandemics.

Our study has a few limitations. First, the analysis data only contains information about people who were enumerated during the 2011 Scottish Census and recorded in the CHI register as of 1 March 2020. Therefore, the disaggregated analyses excluded an estimated 6% of people who were living in Scotland in 2011 but did not take part in the 2011 census. We also excluded census respondents who could not be linked to the EAVE-II data, and people who emigrated since 2011.^14 15^ We also only considered adults (aged>16 years) in our analyses because COVID-19 outcomes tend to be less severe in children.^28^ Second, out-migration may not have been fully captured through the CHI register, since we are only able to look at recorded infections and not undiagnosed. The existence of redundant and historic CHIs inflated the number of individuals in the cohort, which may have led to biased estimates. In addition, we may have failed to capture migrants who were not registered with a general practice due to a lack of documentation. Third, there is the potential of selection bias in the estimated risks caused by people who did not take part in the census, those who did not link to the census (ie, linkage bias) and recent migrants who did not participate in the 2011 census.^29^ Finally, the risk of positive SARS-CoV-2 test represents the risk of testing positive, which may be susceptible to bias due to ethnic differences in the use of the testing system. In turn, this may have led to biased estimates of the risk of hospitalisation or death following a confirmed positive SARS-CoV-2 test. However, despite these limitations, this paper presents a comprehensive analysis of inequalities faced by ethnic minorities during the COVID-19 pandemic in Scotland.

## Conclusions

In conclusion, the risks of COVID-19-related outcomes were higher in some ethnic minority groups in Scotland, in particular among the White Gypsy/Traveller and Pakistani groups. Ethnic minority groups experienced higher risks of severe COVID-19 even after restricting to confirmed infections, indicating that community care is a contributing factor to these inequalities. However, inequalities in COVID-19-related deaths were not observed following hospitalisation, suggesting the provision of secondary care did not contribute to ethnic variations. We recommend future research to understand mediating pathways that generate these ethnic inequalities in COVID-19.

## Data Availability

Data may be obtained from a third party and are not publicly available. Under the provisions of the Statistics and Registration Service Act 2007, the linked 2011 census data used in this study are not permitted to be shared.
