# Supplementary material for: Ethnic inequalities in positive SARS-CoV-2 tests, infection prognosis, COVID-19 hospitalisations and deaths: analysis of 2 years of a record linked national cohort study in Scotland
Source: J Epidemiol Community Health. 2023 Jul 31;77(10):641–8. doi: 10.1136/jech-2023-220501 (PMC10511958; doi:10.1136/jech-2023-220501)
Supplement: Supplementary data [file jech-2023-220501supp001.pdf]

Supplementary material: Ethnic inequalities in positive SARS-CoV-2 tests, infection prognosis, COVID-19 hospitalisations, and deaths: Analysis of two years of a record linked national cohort study in Scotland

Table S1: Ethnic differences in the risk of COVID-19 related hospitalisations and deaths, hospitalisations, deaths and positive SARS-CoV-2 tests based on 5 aggregated ethnic categories across all waves.

Variable	Categories	Hospitalisations or deaths		Hospitalisations		Deaths		Positive SARS-CoV-2 tests	
		Age-sex adjusted	Plus health board	Age-sex adjusted	Plus health board	Age-sex adjusted	Plus health board	Age-sex adjusted	Plus health board
		HR (95% CI)	HR (95% CI)	HR (95% CI)	HR (95% CI)	HR (95% CI)	HR (95% CI)	HR (95% CI)	HR (95% CI)
Ethnic group	White	1(ref)	1(ref)	1(ref)	1(ref)	1(ref)	1(ref)	1(ref)	1(ref)
	Mixed	1.05 (0.85-1.31)	1.04 (0.84-1.29)	1.07 (0.86-1.34)	1.07 (0.86-1.33)	0.90 (0.50-1.63)	0.89 (0.49-1.60)	0.80 (0.78-0.82)	0.79 (0.77-0.81)
	Asian	1.37 (1.28-1.46)	1.26 (1.18-1.34)	1.40 (1.31-1.50)	1.30 (1.21-1.39)	1.36 (1.18-1.57)	1.19 (1.03-1.38)	0.80 (0.79-0.81)	0.77 (0.76-0.78)
	Black or African	1.37 (1.19-1.59)	1.35 (1.17-1.56)	1.44 (1.24-1.67)	1.43 (1.23-1.65)	0.60 (0.33-1.08)	0.57 (0.32-1.04)	0.90 (0.88-0.92)	0.88 (0.87-0.90)
	Other	1.26 (1.06-1.50)	1.25 (1.05-1.48)	1.32 (1.11-1.57)	1.31 (1.10-1.57)	0.49 (0.25-0.98)	0.48 (0.24-0.95)	0.77 (0.75-0.79)	0.76 (0.75-0.78)
Age	16-20	Ref	Ref	Ref	Ref	Ref	Ref	1 (Ref)	1 (Ref)
	21-25	1.23 (1.06-1.42)	1.23 (1.06-1.42)	1.23 (1.07-1.43)	1.23 (1.07-1.43)	1.33 (0.52-3.38)	1.33 (0.52-3.37)	0.91 (0.90-0.92)	0.91 (0.90-0.92)
	26-30	1.65 (1.44-1.89)	1.65 (1.44-1.89)	1.67 (1.45-1.91)	1.66 (1.45-1.91)	1.05 (0.40-2.75)	1.04 (0.40-2.73)	0.80 (0.79-0.81)	0.80 (0.79-0.81)
	31-35	2.01 (1.76-2.29)	2.00 (1.76-2.29)	2.02 (1.76-2.30)	2.01 (1.76-2.30)	2.55 (1.10-5.89)	2.53 (1.09-5.85)	0.73 (0.73-0.74)	0.73 (0.73-0.74)
	36-40	2.55 (2.24-2.90)	2.55 (2.24-2.90)	2.54 (2.23-2.89)	2.54 (2.23-2.90)	4.68 (2.11-10.39)	4.66 (2.10-10.34)	0.74 (0.73-0.74)	0.74 (0.73-0.74)
	41-45	3.22 (2.84-3.66)	3.23 (2.85-3.67)	3.18 (2.80-3.62)	3.19 (2.81-3.63)	9.06 (4.18-19.63)	9.06 (4.18-19.63)	0.70 (0.69-0.70)	0.70 (0.69-0.70)
	46-50	4.10 (3.62-4.63)	4.10 (3.62-4.64)	4.03 (3.56-4.56)	4.03 (3.56-4.57)	16.55 (7.76-35.27)	16.55 (7.76-35.26)	0.59 (0.59-0.60)	0.59 (0.59-0.60)
	51-55	5.53 (4.90-6.23)	5.52 (4.89-6.22)	5.42 (4.80-6.12)	5.41 (4.79-6.11)	24.12 (11.38-51.09)	24.06 (11.36-50.97)	0.52 (0.52-0.53)	0.52 (0.52-0.53)
	56-60	7.15 (6.35-8.05)	7.15 (6.35-8.05)	6.96 (6.17-7.85)	6.96 (6.17-7.85)	43.36 (20.56-91.48)	43.33 (20.54-91.41)	0.44 (0.43-0.44)	0.44 (0.43-0.44)
	61-65	8.89 (7.90-10.01)	8.94 (7.94-10.07)	8.51 (7.55-9.59)	8.55 (7.59-9.64)	75.17 (35.70-158.26)	75.64 (35.92-159.25)	0.32 (0.32-0.33)	0.32 (0.32-0.33)
	66-70	11.32 (10.06-12.74)	11.51 (10.22-12.95)	10.68 (9.48-12.04)	10.85 (9.62-12.22)	119.44 (56.78-251.25)	121.80 (57.90-256.21)	0.22 (0.22-0.22)	0.22 (0.22-0.23)
	71-75	16.70 (14.85-18.78)	17.12 (15.23-19.25)	15.16 (13.47-17.07)	15.53 (13.79-17.48)	228.58 (108.79-480.27)	235.47 (112.07-494.74)	0.19 (0.19-0.19)	0.19 (0.19-0.20)
	76-80	26.15 (23.26-29.40)	26.82 (23.85-30.14)	22.46 (19.95-25.29)	23.00 (20.43-25.90)	414.19 (197.19-869.99)	426.97 (203.27-896.82)	0.20 (0.20-0.20)	0.20 (0.20-0.20)
	81-85	41.26 (36.71-46.37)	42.20 (37.55-47.42)	32.84 (29.17-36.97)	33.56 (29.81-37.78)	795.32 (378.76-1670.01)	816.54 (388.87-1714.57)	0.24 (0.23-0.24)	0.24 (0.23-0.24)
	86-90	61.73 (54.89-69.42)	63.40 (56.38-71.31)	43.28 (38.38-48.80)	44.44 (39.41-50.11)	1423.82 (678.04-2989.90)	1467.02 (698.61-3080.61)	0.31 (0.30-0.32)	0.31 (0.31-0.32)
	>90	75.50 (66.97-85.12)	77.90 (69.09-87.83)	40.96 (36.11-46.46)	42.27 (37.26-47.95)	2169.62 (1032.81-4557.71)	2245.65 (1069.00-4717.43)	0.37 (0.36-0.38)	0.37 (0.36-0.38)
Sex	Male	Ref	Ref	Ref	Ref	Ref	Ref	Ref	Ref
	Female	0.80 (0.79-0.82)	0.80 (0.78-0.81)	0.80 (0.79-0.82)	0.80 (0.78-0.81)	0.68 (0.65-0.70)	0.67 (0.64-0.69)	1.14 (1.14-1.15)	1.14 (1.14-1.15)
Health Board	Ayrshire and Arran		Ref		Ref		Ref		Ref
	Borders		0.57 (0.53-0.62)		0.58 (0.53-0.63)		0.56 (0.49-0.64)		0.74 (0.72-0.75)
	Dumfries and Galloway		0.57 (0.53-0.61)		0.58 (0.54-0.63)		0.43 (0.38-0.49)		0.80 (0.78-0.81)
	Forth Valley		0.94 (0.89-0.98)		0.91 (0.87-0.96)		0.95 (0.87-1.03)		1.00 (0.99-1.01)
	Grampian		0.47 (0.44-0.49)		0.43 (0.41-0.46)		0.50 (0.46-0.54)		0.73 (0.72-0.74)
	Highland		0.42 (0.39-0.44)		0.38 (0.36-0.41)		0.40 (0.36-0.45)		0.71 (0.71-0.72)
	Lothian		0.69 (0.66-0.72)		0.64 (0.62-0.67)		0.76 (0.71-0.82)		0.88 (0.87-0.89)
	Orkney		0.28 (0.22-0.35)		0.28 (0.22-0.36)		0.21 (0.13-0.34)		0.44 (0.42-0.46)
	Shetland		0.21 (0.15-0.27)		0.18 (0.13-0.25)		0.29 (0.19-0.45)		0.37 (0.35-0.39)
	Western Isles		0.45 (0.38-0.54)		0.48 (0.40-0.57)		0.32 (0.22-0.45)		0.66 (0.64-0.68)
	Fife		0.66 (0.62-0.69)		0.63 (0.60-0.66)		0.67 (0.61-0.73)		0.89 (0.88-0.90)
	Tayside		0.67 (0.63-0.70)		0.63 (0.60-0.67)		0.72 (0.66-0.78)		0.89 (0.88-0.90)

Amele S, *et al. J Epidemiol Community Health* 2023; 77:641–648. doi: 10.1136/jech-2023-220501

	Greater Glasgow and Clyde		1.04 (1.00-1.08)		1.00 (0.96-1.04)		1.15 (1.08-1.22)		1.09 (1.08-1.10)
	Lanarkshire		1.11 (1.07-1.15)		1.09 (1.04-1.13)		1.19 (1.11-1.27)		1.17 (1.16-1.18)

HR (95%CI) = Hazard ratio (95% Confidence Interval)

Table S2: Ethnic differences in the risk of COVID-19 related hospitalisations and deaths, hospitalisations, deaths and positive SARS-CoV-2 tests based on 16 disaggregated ethnic subcategories across all waves.

**Variable**	**Categories**	**Hospitalisations and deaths**		**Hospitalisations**		**Deaths**		**Positive SARS-CoV-2 tests**	
		Age-sex adjusted	Plus health board	Age-sex adjusted	Plus health board	Age-sex adjusted	Plus health board	Age-sex adjusted	Plus health board
		HR (95% CI)	HR (95% CI)	HR (95% CI)	HR (95% CI)	HR (95% CI)	HR (95% CI)	HR (95% CI)	HR (95% CI)
Ethnic group	White Scottish	1(ref)	1(ref)	1(ref)	1(ref)	1(ref)	1(ref)	1(ref)	1(ref)
	White Other British	0.58 (0.55-0.60)	0.67 (0.64-0.71)	0.57 (0.54-0.60)	0.67 (0.64-0.71)	0.59 (0.55-0.64)	0.71 (0.66-0.77)	0.71 (0.70-0.71)	0.77 (0.76-0.78)
	White Irish	0.91 (0.83-1.01)	0.83 (0.76-0.92)	0.90 (0.81-1.00)	0.82 (0.74-0.92)	0.90 (0.77-1.06)	0.80 (0.68-0.93)	1.01 (0.99-1.04)	0.98 (0.96-1.00)
	White Gypsy/Traveller	2.15 (1.55-2.96)	2.30 (1.67-3.17)	2.34 (1.69-3.25)	2.52 (1.82-3.50)	1.99 (1.03-3.82)	2.10 (1.09-4.04)	0.76 (0.70-0.83)	0.79 (0.73-0.86)
	White Polish	0.85 (0.72-1.02)	0.93 (0.78-1.11)	0.87 (0.72-1.04)	0.96 (0.80-1.15)	0.82 (0.53-1.28)	0.87 (0.56-1.35)	0.92 (0.91-0.94)	0.97 (0.95-0.99)
	Other White	0.66 (0.58-0.74)	0.71 (0.62-0.80)	0.66 (0.58-0.75)	0.71 (0.62-0.81)	0.66 (0.52-0.83)	0.69 (0.55-0.86)	0.68 (0.67-0.70)	0.71 (0.70-0.73)
	Mixed or multiple ethnic groups	0.79 (0.57-1.10)	0.82 (0.59-1.14)	0.77 (0.54-1.09)	0.80 (0.56-1.14)	1.19 (0.66-2.15)	1.24 (0.69-2.25)	0.79 (0.76-0.82)	0.81 (0.78-0.84)
	Pakistani Pakistani Scottish or Pakistani British	2.41 (2.19-2.65)	2.11 (1.92-2.32)	2.49 (2.26-2.75)	2.20 (1.99-2.43)	2.35 (1.95-2.84)	1.99 (1.65-2.41)	1.06 (1.04-1.08)	0.97 (0.95-0.99)
	Indian Indian Scottish or Indian British	0.89 (0.73-1.09)	0.82 (0.67-1.00)	0.91 (0.74-1.12)	0.85 (0.69-1.04)	0.88 (0.60-1.29)	0.77 (0.52-1.13)	0.89 (0.86-0.92)	0.86 (0.83-0.89)
	Bangladeshi Bangladeshi Scottish or Bangladeshi British	1.66 (1.06-2.60)	1.84 (1.18-2.89)	1.75 (1.11-2.79)	2.00 (1.26-3.18)	1.07 (0.35-3.32)	1.09 (0.35-3.40)	0.84 (0.77-0.92)	0.90 (0.83-0.98)
	Chinese Chinese Scottish or Chinese British	0.67 (0.53-0.85)	0.65 (0.51-0.82)	0.62 (0.48-0.81)	0.60 (0.46-0.79)	1.10 (0.77-1.57)	1.03 (0.73-1.47)	0.43 (0.41-0.45)	0.43 (0.41-0.45)
	Other Asian	1.26 (1.00-1.58)	1.29 (1.03-1.63)	1.30 (1.02-1.64)	1.35 (1.06-1.70)	1.54 (0.97-2.44)	1.53 (0.96-2.43)	0.88 (0.85-0.91)	0.90 (0.87-0.93)
	African	1.42 (1.14-1.78)	1.41 (1.13-1.77)	1.51 (1.20-1.89)	1.51 (1.21-1.89)	0.64 (0.29-1.44)	0.62 (0.28-1.37)	0.84 (0.81-0.87)	0.82 (0.79-0.85)
	Caribbean or Black	0.87 (0.54-1.40)	0.90 (0.56-1.45)	0.92 (0.56-1.50)	0.96 (0.59-1.57)	1.01 (0.42-2.44)	1.04 (0.43-2.50)	0.86 (0.80-0.92)	0.86 (0.80-0.92)
	Arab Arab Scottish or Arab British	1.41 (1.01-1.97)	1.38 (0.99-1.94)	1.46 (1.03-2.07)	1.45 (1.02-2.05)	0.90 (0.37-2.15)	0.87 (0.36-2.09)	0.76 (0.71-0.80)	0.73 (0.69-0.78)
	Other Ethnic Group	1.09 (0.71-1.67)	1.08 (0.71-1.66)	1.16 (0.75-1.79)	1.16 (0.75-1.79)	0.6 (0.20-1.95)	0.61 (0.20-1.91)	0.78 (0.72-0.85)	0.77 (0.72-0.84)
Age	16-20	Ref	Ref	Ref	Ref	Ref	Ref	Ref	Ref
	21-25	1.27 (1.08-1.49)	1.27 (1.08-1.49)	1.28 (1.08-1.50)	1.27 (1.08-1.50)	1.39 (0.55-3.52)	1.38 (0.54-3.50)	0.93 (0.92-0.94)	0.93 (0.92-0.94)
	26-30	1.70 (1.46-1.99)	1.69 (1.45-1.97)	1.72 (1.47-2.01)	1.71 (1.46-1.99)	1.16 (0.44-3.05)	1.15 (0.44-3.02)	0.80 (0.80-0.81)	0.81 (0.80-0.81)
	31-35	2.09 (1.80-2.43)	2.08 (1.79-2.41)	2.10 (1.80-2.44)	2.08 (1.79-2.42)	2.94 (1.27-6.79)	2.91 (1.26-6.72)	0.73 (0.72-0.74)	0.73 (0.72-0.74)
	36-40	2.50 (2.16-2.89)	2.48 (2.15-2.87)	2.48 (2.14-2.88)	2.47 (2.13-2.86)	5.23 (2.36-11.60)	5.19 (2.34-11.50)	0.73 (0.73-0.74)	0.73 (0.72-0.74)
	41-45	3.08 (2.67-3.55)	3.08 (2.67-3.55)	3.02 (2.62-3.49)	3.02 (2.61-3.49)	9.61 (4.44-20.82)	9.58 (4.42-20.75)	0.70 (0.69-0.71)	0.70 (0.69-0.71)
	46-50	4.06 (3.54-4.65)	4.05 (3.53-4.64)	3.97 (3.46-4.56)	3.96 (3.45-4.55)	16.7 (7.84-35.60)	16.65 (7.81-35.48)	0.60 (0.59-0.60)	0.60 (0.59-0.60)
	51-55	5.52 (4.83-6.31)	5.50 (4.81-6.28)	5.39 (4.71-6.17)	5.37 (4.69-6.15)	23.54 (11.11-49.87)	23.41 (11.05-49.60)	0.52 (0.52-0.53)	0.53 (0.52-0.53)
	56-60	7.22 (6.33-8.23)	7.20 (6.31-8.21)	7.01 (6.13-8.01)	6.99 (6.11-7.98)	41.55 (19.70-87.65)	41.35 (19.60-87.24)	0.43 (0.43-0.44)	0.44 (0.43-0.44)
	61-65	9.13 (8.01-10.41)	9.14 (8.02-10.42)	8.70 (7.62-9.93)	8.71 (7.62-9.94)	70.73 (33.59-148.91)	70.76 (33.61-148.98)	0.32 (0.32-0.32)	0.32 (0.32-0.33)
	66-70	11.80 (10.35-13.45)	11.92 (10.46-13.59)	11.10 (9.73-12.67)	11.21 (9.82-12.80)	110.90 (52.72-233.28)	112.24 (53.36-236.10)	0.22 (0.22-0.22)	0.23 (0.22-0.23)
	71-75	17.68 (15.53-20.12)	17.98 (15.80-20.47)	15.99 (14.03-18.24)	16.26 (14.26-18.54)	210.80 (100.33-	215.09 (102.38-	0.19 (0.19-0.19)	0.20 (0.19-0.20)

						442.90)	451.92)		
	76-80	27.75 (24.39-31.58)	28.29 (24.86-32.20)	3.74 (20.82-27.06)	24.18 (21.21-27.57)	377.57 (179.76-793.06)	386.32 (183.92-811.44)	0.20 (0.19-0.20)	0.21 (0.20-0.21)
	81-85	43.78 (38.48-49.81)	44.61 (39.21-50.75)	34.63 (30.37-39.48)	35.28 (30.94-40.22)	720.33 (343.05-1512.52)	735.68 (350.36-1544.76)	0.23 (0.23-0.24)	0.25 (0.24-0.25)
	86-90	65.78 (57.78-74.89)	67.38 (59.18-76.71)	45.79 (40.11-52.28)	46.93 (41.10-53.58)	1282.93 (610.96-2693.99)	1316.02 (626.71-2763.49)	0.31 (0.30-0.32)	0.33 (0.32-0.33)
	>90	81.04 (71.02-92.46)	83.28 (72.99-95.03)	43.69 (38.06-50.16)	(44.9439.15-51.60)	1947.17 (926.94-4090.32)	2004.42 (954.18-4210.61)	0.37 (0.36-0.38)	0.38 (0.37-0.39)
Sex	Male	Ref	Ref	Ref	Ref	Ref	Ref	Ref	Ref
	Female	0.80 (0.79-0.82)	0.80 (0.78-0.81)	0.80 (0.79-0.82)	0.80 (0.78-0.81)	0.68 (0.65-0.70)	0.67 (0.6540.69)	1.15 (1.15-1.16)	1.15 (1.15-1.16)
Health Board	Ayrshire and Arran		Ref		Ref		Ref		Ref
	Borders		0.61 (0.56-0.66)		0.62 (0.57-0.67)		0.57 (0.50-0.66)		0.75 (0.73-0.76)
	Dumfries and Galloway		0.59 (0.55-0.63)		0.60 (0.56-0.65)		0.46 (0.40-0.52)		0.83 (0.82-0.84)
	Forth Valley		0.94 (0.89-0.98)		0.91 (0.86-0.96)		0.94 (0.86-1.02)		1.00 (0.99-1.01)
	Grampian		0.47 (0.45-0.49)		0.43 (0.41-0.46)		0.50 (0.46-0.55)		0.73 (0.73-0.74)
	Highland		0.42 (0.40-0.45)		0.38 (0.35-0.41)		0.41 (0.37-0.46)		0.72 (0.71-0.73)
	Lothian		0.71 (0.68-0.74)		0.66 (0.63-0.69)		0.77 (0.72-0.83)		0.90 (0.89-0.91)
	Orkney		0.29 (0.23-0.38)		0.30 (0.23-0.39)		0.22 (0.13-0.35)		0.46 (0.44-0.48)
	Shetland		0.21 (0.16-0.28)		0.18 (0.13-0.26)		0.29 (0.19-0.45)		0.38 (0.36-0.40)
	Western Isles		0.45 (0.38-0.54)		0.48 (0.39-0.57)		0.32 (0.22-0.45)		0.69 (0.66-0.71)
	Fife		0.66 (0.63-0.70)		0.63 (0.59-0.67)		0.67 (0.61-0.73)		0.91 (0.90-0.92)
	Tayside		0.67 (0.64-0.70)		0.63 (0.60-0.67)		0.72 (0.67-0.78)		0.89 (0.88-0.89)
	Greater Glasgow and Clyde		1.03 (0.99-1.07)		0.98 (0.94-1.02)		1.15 (1.08-1.23)		1.10 (1.09-1.11)
	Lanarkshire		1.10 (1.05-1.14)		1.07 (1.03-1.12)		1.16 (1.09-1.25)		1.16 (1.15-1.17)

HR (95%CI) = Hazard ratio (95% Confidence Interval)

Amele S, *et al. J Epidemiol Community Health* 2023; 77:641–648. doi: 10.1136/jech-2023-220501

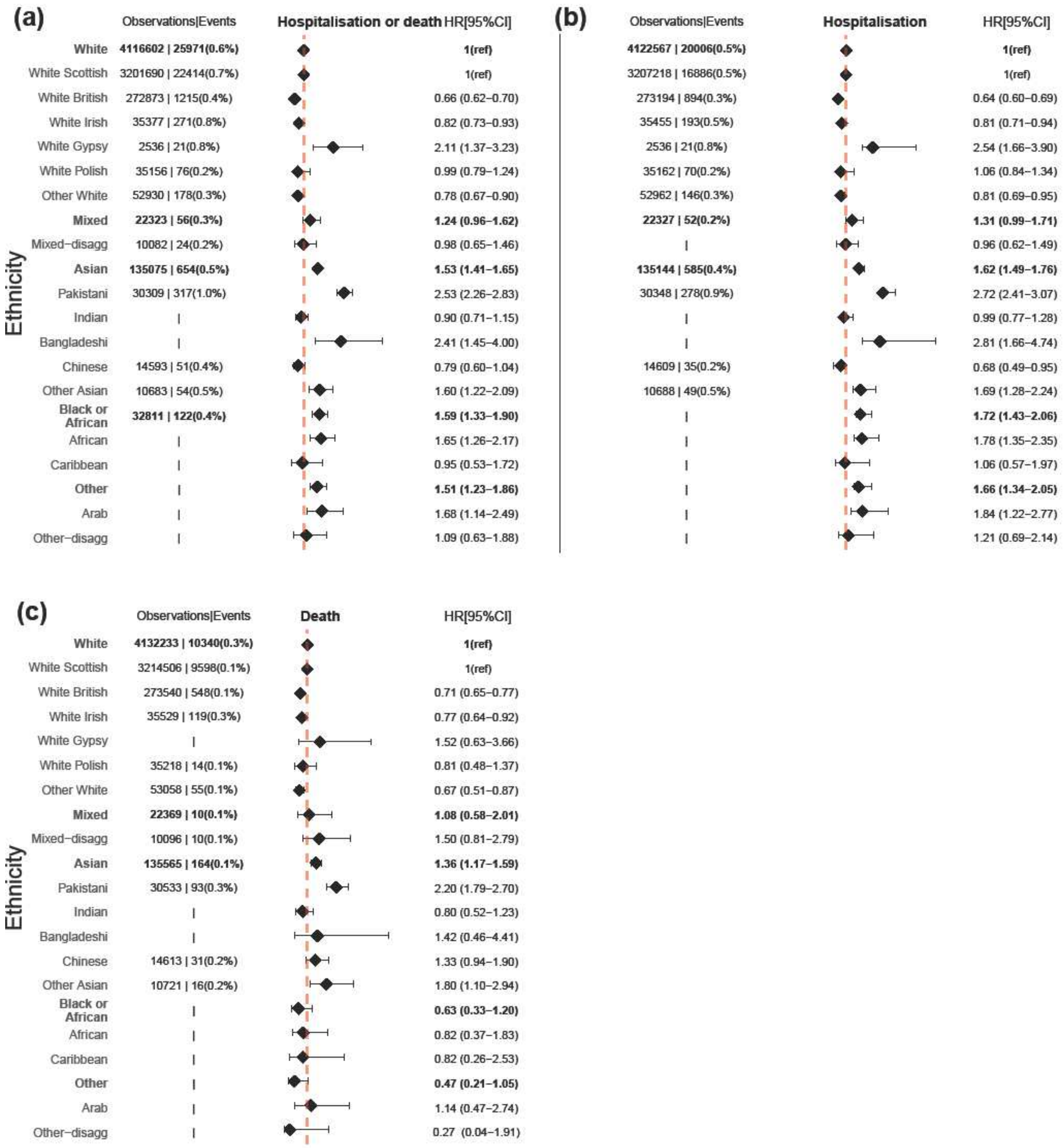

Figure S1: Ethnic differences in the risk of COVID-19 related outcomes for: (a) hospitalisations or deaths, (b) hospitalisation, and (c) deaths where primay position of hospitalisation was only COVID-19.

Models adjusted for age, sex and health board. Bold estimates are for aggregated ethic groups with White group as the reference category. The non-bold estimates are for disaggregated ethnic groups with White Scottish as the reference category. Data are observations and events, HR (95%CI) = Hazard ratio (95% Confidence Interval. The bold and non-bold HR estimates are not comparable since they are based on different reference categories. Observations and Events for ethnic groups containing small numbers (≤10) were removed as per data disclosure agreements and indicated by only low numbers not allowable for disclosure release are represented by |.

Amele S, *et al. J Epidemiol Community Health* 2023; 77:641–648. doi: 10.1136/jech-2023-220501

Table S3: Ethnic differences in the risk of COVID-19 related hospitalisations or deaths, hospitalisations and deaths based on 5 aggregated ethnic categories across all waves where primay position of hospitalisation was only COVID-19.

Variable	Categories	Hospitalisations or deaths		Hospitalisations		Deaths	
		Age-sex adjusted	Plus health board	Age-sex adjusted	Plus health board	Age-sex adjusted	Plus health board
		HR (95% CI)	HR (95% CI)	HR (95% CI)	HR (95% CI)	HR (95% CI)	HR (95% CI)
Ethnic group	White	1(ref)	1(ref)	1(ref)	1(ref)	1(ref)	1(ref)
	Mixed	1.25 (0.96-1.63)	1.24 (0.96-1.62)	1.31 (0.99-1.72)	1.31 (0.99-1.71)	1.10 (0.59-2.05)	1.08 (0.58-2.01)
	Asian	1.68 (1.55-1.81)	1.53 (1.41-1.65)	1.75 (1.61-1.91)	1.62 (1.49-1.76)	1.57 (1.35-1.84)	1.36 (1.17-1.59)
	Black or African	1.62 (1.36-1.94)	1.59 (1.33-1.90)	1.73 (1.44-2.07)	1.72 (1.43-2.06)	0.66 (0.34-1.27)	0.63 (0.33-1.20)
	Other	1.54 (1.25-1.89)	1.51 (1.23-1.86)	1.67 (1.35-2.06)	1.66 (1.34-2.05)	0.49 (0.22-1.10)	0.47 (0.21-1.05)
Age	16-20	Ref	Ref	Ref	Ref	Ref	Ref
	21-25	1.81 (1.41-2.32)	1.81 (1.41-2.31)	1.80 (1.41-2.31)	1.80 (1.41-2.31)	2.33 (0.24-22.38)	2.32 (0.24-22.28)
	26-30	2.59 (2.05-3.28)	2.58 (2.05-3.27)	2.58 (2.04-3.27)	2.58 (2.04-3.26)	5.87 (0.73-46.90)	5.82 (0.73-46.52)
	31-35	3.12 (2.48-3.93)	3.11 (2.48-3.91)	3.10 (2.46-3.90)	3.10 (2.46-3.90)	10.71 (1.41-81.06)	10.61 (1.40-80.34)
	36-40	4.41 (3.53-5.52)	4.41 (3.53-5.51)	4.32 (3.45-5.41)	4.33 (3.46-5.42)	22.59 (3.08-165.48)	22.46 (3.07-164.51)
	41-45	6.15 (4.93-7.66)	6.17 (4.95-7.68)	5.92 (4.74-7.39)	5 .94 (4.76-7.42)	50.65 (7.02-365.18)	50.63 (7.02-365.08)
	46-50	7.97 (6.42-9.89)	7.97 (6.43-9.89)	7.71 (6.20-9.58)	7.71 (6.21-9.59)	76.16 (10.63-545.67)	76.13 (10.62-545.44)
	51-55	11.42 (9.23-14.13)	11.39 (9.21-14.09)	11.00 (8.88-13.63)	10.97 (8.86-13.59)	126.42 (17.72-902.02)	126.06 (17.67-899.43)
	56-60	15.08 (12.21-18.63)	15.07 (12.20-18.61)	14.17 (11.45-17.52)	14.14 (11.43-17.49)	233.61 (32.81-1663.10)	233.27 (32.77-1660.72)
	61-65	18.54 (15.02-22.90)	18.63 (15.09-23.01)	17.04 (13.77-21.07)	17.09 (13.82-21.14)	402.10 (56.54-2859.89)	404.44 (56.86-2876.51)
	66-70	23.83 (19.31-29.42)	24.22 (19.62-29.90)	21.31 (17.23-26.35)	21.61 (17.47-26.72)	623.68 (87.73-4433.96)	636.28 (89.50-4523.53)
	71-75	35.64 (28.90-43.95)	36.55 (29.64-45.07)	30.26 (24.49-37.38)	30.94 (25.05-38.22)	1225.97 (172.55-8710.47)	1264.18 (177.93-8981.95)
	76-80	56.09 (45.49-69.16)	57.52 (46.65-70.92)	43.46 (35.18-53.69)	44.43 (35.96-54.89)	2227.27 (313.53-15822.36)	2298.50 (323.55-16328.36)
	81-85	90.52 (73.43-111.57)	92.52 (75.06-114.04)	62.67 (50.73-77.43)	63.87 (51.70-78.92)	4329.10 (609.50-30748.49)	4447.31 (626.14-31588.08)
	86-90	138.01 (111.90-170.21)	141.67 (114.87-174.72)	78.73 (63.61-97.43)	80.67 (65.19-99.84)	7870.60 (1108.09-55903.80)	8109.55 (1141.73-57601.02)
	>90	179.18 (145.04-221.37)	184.81 (149.59-228.32)	70.13 (56.29-87.37)	72.24 (57.98-90.00)	12341.53 (1737.27-87673.79)	12775.48 (1798.36-90756.62)
Sex	Male	Ref	Ref	Ref	Ref	Ref	Ref
	Female	0.79 (0.77-0.81)	0.78 (0.76-0.80)	0.80 (0.79-0.82)	0.79 (0.77-0.82)	0.79 (0.77-0.81)	0.65 (0.62-0.67)
Health Board	Ayrshire and Arran		Ref		Ref		Ref
	Borders		0.63 (0.57-0.70)		0.68 (0.61-0.76)		0.56 (0.48-0.65)
	Dumfries and Galloway		0.59 (0.55-0.65)		0.64 (0.59-0.71)		0.42 (0.36-0.49)
	Forth Valley		1.11 (1.05-1.18)		1.14 (1.07-1.22)		0.94 (0.85-1.03)
	Grampian		0.51 (0.48-0.54)		0.48 (0.45-0.52)		0.51 (0.46-0.57)
	Highland		0.41 (0.38-0.44)		0.38 (0.34-0.41)		0.39 (0.35-0.45)
	Lothian		0.68 (0.65-0.72)		0.61 (0.57-0.65)		0.78 (0.72-0.85)
	Orkney		0.23 (0.16-0.32)		0.26 (0.18-0.38)		0.12 (0.06-0.25)
	Shetland		0.24 (0.17-0.33)		0.19 (0.12-0.29)		0.31 (0.19-0.50)
	Western Isles		0.42 (0.33-0.53)		0.48 (0.38-0.61)		0.25 (0.16-0.39)
	Fife		0.65 (0.61-0.69)		0.61 (0.56-0.65)		0.67 (0.60-0.74)
	Tayside		0.74 (0.70-0.78)		0.72 (0.67-0.77)		0.74 (0.67-0.81)
	Greater Glasgow and Clyde		1.13 (1.08-1.18)		1.08 (1.02-1.13)		1.20 (1.11-1.29)
	Lanarkshire		1.15 (1.10-1.21)		1.12 (1.06-1.19)		1.19 (1.10-1.29)

HR (95%CI) = Hazard ratio (95% Confidence Interval)

Table S4: Ethnic differences in the risk of COVID-19 related hospitalisations and deaths, hospitalisations, deaths, and positive SARS-CoV-2 tests based on 16 disaggregated ethnic subcategories across all waves where primay position of hospitalisation was only COVID-19.

Variable	Categories	Hospitalisations or deaths		Hospitalisations		Deaths	
		Age-sex adjusted	Plus health board	Age-sex adjusted	Plus health board	Age-sex adjusted	Plus health board
		HR (95% CI)	HR (95% CI)	HR (95% CI)	HR (95% CI)	HR (95% CI)	HR (95% CI)
Ethnic group	White Scottish	1(ref)	1(ref)	1(ref)	1(ref)	1(ref)	1(ref)
	White Other British	0.56 (0.53-0.59)	0.66 (0.62-0.70)	0.55 (0.51-0.58)	0.64 (0.60-0.69)	0.59 (0.54-0.64)	0.71 (0.65-0.77)
	White Irish	0.91 (0.81-1.02)	0.82 (0.73-0.93)	0.89 (0.77-1.02)	0.81 (0.71-0.94)	0.88 (0.73-1.05)	0.77 (0.64-0.92)
	White Gypsy/Traveller	1.98 (1.29-3.04)	2.11 (1.37-3.23)	2.38 (1.55-3.65)	2.54 (1.66-3.90)	1.44 (0.60-3.47)	1.52 (0.63-3.66)
	White Polish	0.91 (0.72-1.14)	0.99 (0.79-1.24)	0.95 (0.75-1.21)	1.06 (0.84-1.34)	0.76 (0.45-1.29)	0.81 (0.48-1.37)
	Other White	0.72 (0.62-0.84)	0.78 (0.67-0.90)	0.74 (0.63-0.87)	0.81 (0.69-0.95)	0.64 (0.49-0.83)	0.67 (0.51-0.87)
	Mixed - disaggregate	0.93 (0.63-1.39)	0.98 (0.65-1.46)	0.91 (0.58-1.40)	0.96 (0.62-1.49)	1.44 (0.78-2.68)	1.50 (0.81-2.79)
	Pakistani	2.92 (2.61-3.26)	2.53 (2.26-2.83)	3.10 (2.75-3.49)	2.72 (2.41-3.07)	2.63 (2.14-3.23)	2.20 (1.79-2.70)
	Indian	0.99 (0.78-1.26)	0.90 (0.71-1.15)	1.07 (0.83-1.38)	0.99 (0.77-1.28)	0.93 (0.60-1.42)	0.80 (0.52-1.23)
	Bangladeshi	2.17 (1.31-3.60)	2.41 (1.45-4.00)	2.43 (1.44-4.10)	2.81 (1.66-4.74)	1.40 (0.45-4.35)	1.42 (0.46-4.41)
	Chinese	0.82 (0.62-1.08)	0.79 (0.60-1.04)	0.70 (0.50-0.97)	0.68 (0.49-0.95)	1.44 (1.01-2.04)	1.33 (0.94-1.90)
	Other Asian	1.56 (1.20-2.04)	1.60 (1.22-2.09)	1.63 (1.23-2.15)	1.69 (1.28-2.24)	1.83 (1.12-2.98)	1.80 (1.10-2.94)
	African	1.68 (1.28-2.20)	1.65 (1.26-2.17)	1.78 (1.34-2.35)	1.78 (1.35-2.35)	0.87 (0.39-1.93)	0.82 (0.37-1.83)
	Caribbean	0.92 (0.51-1.66)	0.95 (0.53-1.72)	1.00 (0.54-1.87)	1.06 (0.57-1.97)	0.80 (0.26-2.47)	0.82 (0.26-2.53)
	Arab	1.72 (1.16-2.54)	1.68 (1.14-2.49)	1.85 (1.23-2.79)	1.84 (1.22-2.77)	1.18 (0.49-2.84)	1.14 (0.47-2.74)
	Other -disaggregate	1.10 (0.64-1.90)	1.09 (0.63-1.88)	1.21 (0.69-2.13)	1.21 (0.69-2.14)	0.28 (0.04-1.96)	0.27 (0.04-1.91)
Age	16-20	Ref	Ref	Ref	Ref	Ref	Ref
	21-25	1.98 (1.49-2.62)	1.97 (1.49-2.61)	1.97 (1.49-2.61)	1.97 (1.48-2.61)	2.43 (0.25-23.36)	2.42 (0.25-23.23)
	26-30	2.90 (2.22-3.78)	2.87 (2.20-3.75)	2.88 (2.20-3.77)	2.86 (2.19-3.75)	6.51 (0.81-52.09)	6.44 (0.80-51.46)
	31-35	3.47 (2.67-4.51)	3.44 (2.65-4.47)	3.44 (2.64-4.48)	3.41 (2.62-4.45)	12.37 (1.63-93.63)	12.21 (1.61-92.44)
	36-40	4.58 (3.55-5.92)	4.55 (3.53-5.88)	4.44 (3.43-5.75)	4.42 (3.41-5.72)	25.31 (3.45-185.41)	25.06 (3.42-183.58)
	41-45	6.23 (4.85-8.02)	6.23 (4.84-8.01)	5.91 (4.58-7.62)	5.91 (4.58-7.62)	53.76 (7.46-387.61)	53.57 (7.43-386.25)
	46-50	8.30 (6.49-10.62)	8.29 (6.48-10.60)	7.96 (6.21-10.20)	7.95 (6.20-10.19)	76.79 (10.72-550.19)	76.52 (10.68-548.25)
	51-55	12.22 (9.59-15.56)	12.17 (9.55-15.50)	11.69 (9.16-14.92)	11.64 (9.12-14.86)	123.12 (17.26-878.50)	122.44 (17.16-873.64)
	56-60	16.17 (12.71-20.57)	16.12 (12.67-20.50)	15.05 (11.81-19.19)	15.01 (11.77-19.13)	223.27 (31.36-1589.46)	222.06 (31.19-1580.88)
	61-65	20.10 (15.81-25.55)	20.12 (15.82-25.58)	18.29 (14.36-23.31)	18.31 (14.37-23.33)	377.34 (53.05-2683.71)	377.33 (53.05-2683.66)
	66-70	26.23 (20.64-33.34)	26.52 (20.86-33.70)	23.26 (18.26-29.63)	23.49 (18.44-29.92)	578.09 (81.32-4109.81)	585.24 (82.32-4160.65)
	71-75	40.01 (31.51-50.79)	40.73 (32.08-51.71)	33.73 (26.51-42.93)	34.29 (26.95-43.63)	1127.20 (158.65-8008.60)	1151.04 (162.01-8177.98)
	76-80	63.17 (49.76-80.18)	64.44 (50.77-81.80)	48.58 (38.18-61.82)	49.46 (38.87-62.95)	2023.76 (284.88-14376.46)	2072.54 (291.75-14723.00)
	81-85	101.69 (80.13-129.04)	103.61 (81.64-131.48)	69.67 (54.74-88.65)	70.88 (55.70-90.19)	3906.22 (549.97-27744.53)	3991.24 (561.93-28348.45)
	86-90	155.49 (122.48-197.42)	159.25 (125.43-202.18)	87.65 (68.77-111.71)	89.75 (70.42-114.38)	7062.89 (994.39-50166.12)	7245.08 (1020.03-51460.30)
	>90	202.83 (159.52-257.90)	208.52 (163.99-265.13)	78.44 (61.17-100.57)	80.65 (62.90-103.41)	11033.28 (1553.14-78379.02)	11358.19 (1598.87-80687.47)
Sex	Male	Ref	Ref	Ref	Ref	Ref	Ref
	Female	0.78 (0.76-0.80)	0.77 (0.75-0.79)	0.79 (0.77-0.81)	0.78 (0.76-0.80)	0.66 (0.63-0.68)	0.65 (0.62-0.67)
Health Board	Ayrshire and Arran		Ref		Ref		Ref
	Borders		0.67 (0.60-0.73)		0.73 (0.65-0.81)		0.57 (0.49-0.67)
	Dumfries and Galloway		0.62 (0.57-0.68)		0.68 (0.61-0.75)		0.45 (0.38-0.52)
	Forth Valley		1.11 (1.04-1.18)		1.15 (1.07-1.23)		0.93 (0.84-1.03)

	Grampian		0.51 (0.48-0.55)		0.48 (0.45-0.52)		0.51 (0.47-0.57)
	Highland		0.42 (0.38-0.45)		0.38 (0.34-0.42)		0.41 (0.36-0.46)
	Lothian		0.70 (0.66-0.74)		0.63 (0.59-0.67)		0.78 (0.72-0.85)
	Orkney		0.24 (0.17-0.34)		0.28 (0.19-0.40)		0.12 (0.06-0.26)
	Shetland		0.25 (0.18-0.35)		0.20 (0.13-0.31)		0.31 (0.19-0.50)
	Western Isles		0.39 (0.31-0.50)		0.45 (0.34-0.58)		0.25 (0.16-0.39)
	Fife		0.65 (0.61-0.70)		0.61 (0.56-0.66)		0.67 (0.61-0.74)
	Tayside		0.74 (0.70-0.79)		0.71 (0.67-0.77)		0.74 (0.68-0.81)
	Greater Glasgow and Clyde		1.12 (1.07-1.18)		1.07 (1.01-1.12)		1.20 (1.11-1.29)
	Lanarkshire		1.14 (1.08-1.20)		1.11 (1.04-1.17)		1.17 (1.08-1.27)

HR (95%CI) = Hazard ratio (95% Confidence Interval)

Table S5: Ethnic differences in the risk of COVID-19 related hospitalisations and deaths, hospitalisations and deaths based on 5 aggregated ethnic categories across waves following a confirmed positive SARS-CoV-2 test and confirmed COVID-19 hospitalisation.

**Variable**	**Categories**	**Confirmed positive SARS-CoV-2 test**						**COVID-19 hospitalisation**	
		**Hospitalisations and deaths**		**Hospitalisations**		**Deaths**		**Deaths**	
		Age-sex adjusted	Plus health board	Age-sex adjusted	Plus health board	Age-sex adjusted	Plus health board	Age-sex adjusted	Plus health board
		HR (95% CI)	HR (95% CI)	HR (95% CI)	HR (95% CI)	HR (95% CI)	HR (95% CI)	HR (95% CI)	HR (95% CI)
Ethnic group	White	1(ref)	1(ref)	1(ref)	1(ref)	1(ref)	1(ref)	1(ref)	1(ref)
	Mixed	1.24 (0.99-1.54)	1.25 (1.00-1.56)	1.28 (1.02-1.61)	1.30 (1.03-1.63)	0.98 (0.53-1.83)	0.97 (0.52-1.81)	0.86 (0.41-1.80)	0.85 (0.40-1.78)
	Asian	1.37 (1.28-1.47)	1.34 (1.25-1.44)	1.42 (1.32-1.52)	1.39 (1.29-1.49)	1.22 (1.05-1.42)	1.17 (1.01-1.37)	1.07 (0.89-1.28)	1.04 (0.87-1.24)
	Black or African	1.46 (1.26-1.70)	1.49 (1.28-1.72)	1.54 (1.32-1.79)	1.57 (1.35-1.82)	0.64 (0.35-1.15)	0.63 (0.35-1.13)	0.58 (0.30-1.11)	0.56 (0.29-1.08)
	Other	1.35 (1.13-1.62)	1.37 (1.14-1.64)	1.46 (1.22-1.74)	1.48 (1.24-1.77)	0.36 (0.16-0.79)	0.63 (0.16-0.79)	0.36 (0.15-0.86)	0.37 (0.15-0.88)
Age	16-20	Ref	Ref	Ref	Ref	Ref	Ref	Ref	Ref
	21-25	1.28 (1.10-1.48)	1.27 (1.10-1.48)	1.28 (1.10-1.49)	1.28 (1.10-1.49)	1.24 (0.44-3.48)	1.23 (0.44-3.47)	1.94 (0.39-9.62)	1.92 (0.39-9.51)
	26-30	1.87 (1.62-2.15)	1.86 (1.61-2.14)	1.88 (1.63-2.17)	1.87 (1.62-2.16)	1.13 (0.39-3.27)	1.13 (0.39-3.25)	0.88 (0.16-4.78)	0.87 (0.16-4.76)
	31-35	2.50 (2.18-2.86)	2.48 (2.17-2.85)	2.49 (2.17-2.86)	2.48 (2.16-2.85)	3.43 (1.40-8.42)	3.41 (1.39-8.36)	2.28 (0.51-10.09)	2.25 (0.51-9.98)
	36-40	3.13 (2.74-3.58)	3.12 (2.73-3.56)	3.13 (2.74-3.58)	3.11 (2.72-3.56)	5.48 (2.31-13.02)	5.45 (2.30-12.94)	3.44 (0.81-14.56)	3.41 (0.81-14.44)
	41-45	4.14 (3.63-4.73)	4.14 (3.63-4.72)	4.08 (3.57-4.65)	4.07 (3.56-4.64)	11.93 (5.18-27.50)	11.88 (5.16-27.38)	5.62 (1.36-23.20)	5.55 (1.35-22.89)
	46-50	5.87 (5.16-6.67)	5.82 (5.13-6.62)	5.77 (5.07-6.56)	5.72 (5.03-6.51)	24.51 (10.82-55.50)	24.34 (10.75-55.11)	9.36 (2.31-37.91)	9.24 (2.28-37.45)
	51-55	8.85 (7.82-10.02)	8.74 (7.72-9.90)	8.68 (7.66-9.84)	8.56 (7.55-9.71)	40.04 (17.80-90.08)	39.61 (17.61-89.10)	10.15 (2.52-40.91)	9.93 (2.46-40.02)
	56-60	13.39 (11.84-15.15)	13.20 (11.67-14.92)	12.96 (11.45-14.67)	12.74 (11.25-14.42)	87.08 (38.89-194.98)	85.91 (38.37-192.36)	15.58 (3.88-62.55)	15.29 (3.81-61.40)
	61-65	21.95 (19.42-24.82)	21.64 (19.14-24.46)	20.81 (18.39-23.56)	20.47 (18.09-23.16)	197.55 (88.41-441.41)	195.11 (87.32-435.95)	22.59 (5.63-90.59)	22.21 (5.54-89.05)
	66-70	39.74 (35.17-44.91)	39.32 (34.79-44.43)	36.73 (32.46-41.56)	36.26 (32.04-41.03)	442.04 (198.01-986.79)	438.46 (196.41-978.80)	29.71 (7.41-119.05)	29.30 (7.31-117.41)
	71-75	69.90 (61.93-78.90)	69.20 (61.31-78.11)	61.47 (54.39-69.48)	60.75 (53.75-68.66)	992.00 (444.92-2211.78)	983.48 (441.10-2192.78)	39.72 (9.92-159.01)	39.19 (9.79-156.93)
	76-80	110.16 (97.61-124.32)	108.58 (96.21-122.54)	90.06 (79.67-101.79)	88.57 (78.35-100.11)	1756.89 (788.19-3916.13)	1735.60 (778.64-3868.69)	43.37 (10.83-173.62)	42.74 (10.67-171.09)
	81-85	153.14 (135.73-172.80)	151.26 (134.06-170.67)	113.09 (100.05-127.85)	111.49 (98.62-126.03)	2998.06 (1345.45-6680.55)	2964.28 (1330.29-6605.30)	53.48 (13.36-214.07)	52.60 (13.14-210.54)
	86-90	178.76 (158.29-201.89)	177.84 (157.56-200.96)	114.50 (101.09-129.68)	113.88 (100.55-128.98)	4316.60 (1937.01-9619.46)	4297.38 (1928.39-9576.64)	63.90 (15.96-255.84)	63.14 (15.77-252.80)

	>90	185.54 (163.79-210.18)	185.77 (163.99-210.45)	89.67 (78.59-102.32)	89.81 (78.71-102.48)	5984.85 (2684.35-13343.44)	5988.09 (2685.80-13350.66)	81.46 (20.33-326.45)	80.32 (20.04-321.91)
Sex	Male	Ref	Ref	Ref	Ref	Ref	Ref	Ref	Ref
	Female	0.78 (0.76-079)	0.78 (0.76-0.79)	0.79 (0.77-0.81)	0.79 (0.77-0.81)	0.65 (0.63-0.68)	0.65 (0.63-0.67)	0.73 (0.70-0.77)	0.73 (0.70-0.77)
Health Board	Ayrshire and Arran		Ref		Ref		Ref		Ref
	Borders		0.82 (0.76-0.90)		0.86 (0.78-0.94)		0.81 (0.70-0.94)		0.95 (0.79-1.13)
	Dumfries and Galloway		0.89 (0.83-0.95)		0.94 (0.87-1.01)		0.67 (0.59-0.78)		0.73 (0.61-0.88)
	Forth Valley		0.88 (0.84-0.93)		0.89 (0.84-0.94)		0.90 (0.82-0.99)		0.90 (0.80-1.01)
	Grampian		0.66 (0.63-0.70)		0.65 (0.61-0.69)		0.68 (0.62-0.75)		0.84 (0.74-0.94)
	Highland		0.68 (0.63-0.72)		0.65 (0.61-0.70)		0.65 (0.58-0.73)		0.66 (0.55-0.78)
	Lothian		0.76 (0.73-0.80)		0.73 (0.70-0.77)		0.84 (0.78-0.90)		0.86 (0.79-0.95)
	Orkney		0.79 (0.61-1.02)		0.83 (0.63-1.09)		0.61 (0.36-1.03)		0.88 (0.46-1.70)
	Shetland		0.69 (0.51-0.93)		0.59 (0.42-0.84)		1.10 (0.70-1.74)		1.72 (0.92-3.21)
	Western Isles		0.69 (0.58-0.83)		0.73 (0.60-0.89)		0.49 (0.35-0.71)		0.66 (0.42-1.04)
	Fife		0.77 (0.73-0.81)		0.76 (0.72-0.81)		0.75 (0.68-0.82)		0.89 (0.79-1.01)
	Tayside		0.83 (0.79-0.87)		0.82 (0.78-0.87)		0.88 (0.81-0.96)		0.94 (0.84-1.05)
	Greater Glasgow and Clyde		0.95 (0.92-0.99)		0.97 (0.93-1.01)		0.95 (0.89-1.02)		1.00 (0.93-1.09)
	Lanarkshire		0.99 (0.95-1.03)		1.00 (0.96-1.05)		0.99 (0.92-1.07)		1.03 (0.94-1.12)

Table S6: Ethnic differences in the risk of COVID-19 related hospitalisations and deaths, hospitalisations and deaths based on 16 disaggregated ethnic subcategories across waves following a confirmed positive SARS-CoV-2 test and COVID-19 hospitalisation.

**Variable**	**Categories**	**Positive SARS-CoV-2 test**						**COVID-19 hospitalisation**	
		**Hospitalisations and deaths**		**Hospitalisations**		**Deaths**		**Deaths**	
		Age-sex adjusted	Plus health board	Age-sex adjusted	Plus health board	Age-sex adjusted	Plus health board	Age-sex adjusted	Plus health board
		HR (95% CI)	HR (95% CI)	HR (95% CI)	HR (95% CI)	HR (95% CI)	HR (95% CI)	HR (95% CI)	HR (95% CI)
Ethnic group	White Scottish	1(ref)	1(ref)	1(ref)	1(ref)	1(ref)	1(ref)	1(ref)	1(ref)
	White Other British	0.80 (0.76-0.84)	0.85 (0.81-0.89)	0.79 (0.75-0.84)	0.84 (0.80-0.89)	0.86 (0.79-0.93)	0.91 (0.84-0.99)	1.02 (0.92-1.13)	1.07 (0.97-1.19)
	White Irish	0.87 (0.79-0.97)	0.84 (0.76-0.93)	0.87 (0.77-0.97)	0.83 (0.74-0.93)	0.86 (0.72-1.02)	0.82 (0.69-0.98)	0.86 (0.68-1.08)	0.83 (0.66-1.05)
	White Gypsy/Traveller	2.52 (1.79-3.54)	2.55 (1.81-3.58)	2.67 (1.88-3.80)	2.72 (1.91-3.86)	2.51 (1.31-4.83)	2.51 (1.30-4.82)	1.50 (0.75-3.00)	1.56 (0.78-3.13)
	White Polish	0.96 (0.80-1.16)	1.03 (0.86-1.23)	0.97 (0.80-1.17)	1.05 (0.87-1.26)	1.02 (0.64-1.63)	1.07 (0.67-1.70)	1.14 (0.65-2.01)	1.18 (0.67-2.09)
	Other White	0.86 (0.75-0.98)	1.03 (0.78-1.01)	0.84 (0.73-0.97)	0.88 (0.77-1.02)	0.87 (0.69-1.12)	0.89 (0.70-1.13)	0.83 (0.61-1.14)	0.84 (0.61-1.15)
	Mixed or multiple ethnic groups	1.01 (0.72-1.42)	1.03 (0.73-1.45)	1.02 (0.71-1.46)	1.04 (0.73-1.49)	1.43 (0.77-2.65)	1.43 (0.77-2.66)	1.69 (0.81-3.56)	1.69 (0.81-3.55)
	Pakistani Pakistani Scottish or Pakistani British	1.81 (1.64-2.00)	1.75 (1.59-1.93)	1.92 (1.73-2.12)	1.84 (1.66-2.04)	1.57 (1.29-1.91)	1.50 (1.23-1.83)	1.21 (0.96-1.52)	1.17 (0.93-1.47)
	Indian Indian Scottish or Indian British	0.92 (0.75-1.13)	0.91 (0.73-1.12)	0.94 (0.75-1.17)	0.92 (0.74-1.15)	0.88 (0.58-1.33)	0.84 (0.56-1.27)	0.93 (0.57-1.52)	0.88 (0.54-1.44)
	Bangladeshi Bangladeshi Scottish or Bangladeshi British	1.55 (0.97-2.50)	1.65 (1.03-2.66)	1.81 (1.13-2.92)	1.99 (1.24-3.20)	0.66 (0.17-2.65)	0.66 (0.16-2.63)	0.64 (0.16-2.58)	0.65 (0.16-2.61)
	Chinese Chinese Scottish or Chinese British	1.28 (0.99-1.65)	1.27 (0.98-1.64)	1.17 (0.88-1.56)	1.17 (0.88-1.55)	**2.11 (1.44-3.08)**	2.05 (1.40-2.98)	1.78 (1.12-2.82)	1.73 (1.09-2.74)
	Other Asian	1.37 (1.08-1.73)	1.41 (1.11-1.78)	1.39 (1.09-1.78)	1.44 (1.13-1.84)	1.75 (1.08-2.81)	1.75 (1.09-2.82)	1.87 (1.11-3.17)	1.83 (1.08-3.10)
	African	1.58 (1.26-1.99)	1.61 (1.28-2.03)	1.70 (1.35-2.15)	1.74 (1.38-2.19)	0.71 (0.32-1.58)	0.70 (0.31-1.56)	0.62 (0.26-1.49)	0.60 (0.25-1.44)

	Caribbean or Black	1.20 (0.75-1.93)	1.22 (0.76-1.96)	1.16 (0.70-1.93)	1.19 (0.72-1.97)	1.68 (0.70-4.04)	1.68 (0.70-4.04)	1.79 (0.67-4.78)	1.76 (0.66-4.70)
	Arab Arab Scottish or Arab British	1.53 (1.08-2.18)	1.54 (1.08-2.19)	1.67 (1.16-2.38)	1.68 (1.18-2.41)	0.79 (0.30-2.12)	0.79 (0.30-2.11)	0.79 (0.25-2.44)	0.80 (0.26-2.48)
	Other Ethnic Group	1.30 (0.84-2.02)	1.31 (0.85-2.04)	0.48 (0.95-2.29)	1.48 (0.96-2.31)	0.45 (0.11-1.82)	0.45 (0.11-1.81)	0.57 (0.14-2.27)	0.59 (0.15-2.35)
Age	16-20	Ref	Ref	Ref	Ref	Ref	Ref	Ref	Ref
	21-25	1.29 (1.09-1.52)	1.28 (1.09-1.52)	1.30 (1.09-1.53)	1.29 (1.09-1.53)	1.26 (0.45-3.54)	1.25 (0.45-3.52)	1.97 (0.40-9.75)	1.95 (0.39-9.66)
	26-30	1.91 (1.63-2.23)	1.89 (1.61-2.22)	1.92 (1.64-2.26)	1.91 (1.63-2.24)	1.25 (0.43-3.60)	1.24 (0.43-3.57)	0.95 (0.17-5.21)	0.95 (0.17-5.21)
	31-35	2.60 (2.23-3.03)	2.58 (2.21-3.01)	2.59 (2.22-3.02)	2.57 (2.20-3.00)	3.95 (1.61-9.70)	3.91 (1.59-9.61)	2.60 (0.59-11.53)	2.57 (0.58-11.40)
	36-40	3.01 (2.59-3.50)	2.99 (2.57-3.47)	3.00 (2.58-3.50)	2.98 (2.56-3.47)	6.11 (2.57-14.49)	6.05 (2.55-14.37)	3.99 (0.94-16.90)	3.96 (0.94-16.77)
	41-45	3.93 (3.39-4.55)	3.91 (3.37-4.53)	3.83 (3.30-4.45)	3.81 (3.29-4.43)	12.56 (5.45-28.93)	12.47 (5.41-28.74)	6.42 (1.56-26.50)	6.36 (1.54-26.26)
	46-50	5.74 (4.98-6.61)	5.69 (4.94-6.56)	5.61 (4.86-6.47)	5.56 (4.82-6.41)	24.52 (10.83-55.52)	24.30 (10.73-55.03)	9.75 (2.40-39.49)	9.63 (2.38-39.01)
	51-55	8.81 (7.68-10.11)	8.71 (7.59-10.00)	8.61 (7.49-9.89)	8.49 (7.39-9.76)	39.02 (17.35-87.79)	38.58 (17.15-86.78)	10.08 (2.50-40.65)	9.88 (2.45-39.85)
	56-60	13.54 (11.82-15.51)	13.36 (11.66-15.30)	13.03 (11.36-14.95)	12.83 (11.19-14.72)	83.45 (37.27-186.85)	82.28 (36.75-184.23)	15.02 (3.74-60.32)	14.77 (3.68-59.32)
	61-65	22.54 (19.69-25.81)	22.24 (19.43-25.46)	21.24 (18.53-24.36)	20.92 (18.24-23.98)	187.21 (83.79-418.30)	184.74 (82.68-412.78)	21.12 (5.27-84.69)	20.80 (5.19-83.43)
	66-70	41.35 (36.13-47.32)	40.93 (35.76-46.84)	38.00 (33.15-43.56)	37.54 (32.75-43.03)	411.29 (184.24-918.15)	407.48 (182.53-909.64)	27.05 (6.75-108.38)	26.72 (6.67-107.10)
	71-75	74.02 (64.76-84.61)	73.32 (64.15-83.81)	64.60 (56.42-73.96)	63.89 (55.80-73.15)	921.45 (413.28-2054.47)	912.50 (409.26-2034.52)	35.43 (8.85-141.87)	35.04 (8.75-140.30)
	76-80	116.3 (102.25-133.53)	115.37 (100.96-131.85)	94.61 (82.63-108.32)	93.23 (81.43-106.74)	1609.97 (722.28-3588.63)	1589.56 (713.12-3543.14)	38.00 (9.49-152.13)	37.53 (9.37-150.25)
	81-85	163.24 (142.88-186.50)	161.56 (141.41-184.59)	118.9 (103.88-136.19)	117.54 (102.66-134.58)	2741.05 (1230.12-6107.84)	2709.22 (1215.83-6036.92)	46.69 (11.66-186.90)	46.01 (11.49-184.19)
	86-90	190.80 (166.86-218.17)	190.04 (166.20-217.31)	120.39 (104.96-138.09)	119.85 (104.49-137.47)	3917.25 (1757.82-8729.48)	3895.21 (1747.92-8680.39)	55.40 (13.84-221.82)	54.85 (13.70-219.62)
	>90	200.35 (174.73-229.73)	200.56 (174.91-229.97)	95.34 (82.55-110.10)	95.49 (82.68-110.28)	5425.99 (2433.70-12097.41)	5419.99 (2431.00-12084.05)	69.43 (17.32-278.28)	68.68 (17.14-275.27)
sex	Male	Ref	Ref	Ref	Ref	Ref	Ref	Ref	Ref
	Female	0.78 (0.77-0.80)	0.78 (0.77-0.80)	0.80 (0.78-0.82)	0.91 (0.83-1.00)	0.65 (0.63-0.68)	0.63 (0.63-0.67)	0.73 (0.70-0.77)	0.73 (0.69-0.76)
Health Board	Ayrshire and Arran		Ref		0.95 (0.87-1.03)		Ref		Ref
	Borders		0.86 (0.79-0.94)		0.89 (0.84-0.95)		0.80 (0.69-0.93)		0.91 (0.76-1.08)
	Dumfries and Galloway		0.89 (0.83-0.96)		0.65 (0.61-0.69)		0.69 (0.60-0.79)		0.74 (0.62-0.88)
	Forth Valley		0.88 (0.84-0.94)		0.65 (0.60-0.70)		0.89 (0.81-0.98)		0.89 (0.79-0.99)
	Grampian		0.66 (0.63-0.70)		0.76 (0.72-0.79)		0.68 (0.62-0.75)		0.83 (0.74-0.94)
	Highland		0.68 (0.64-0.73)		0.86 (0.65-1.13)		0.66 (0.59-0.74)		0.67 (0.56-0.79)
	Lothian		0.78 (0.75-0.82)		0.59 (0.41-0.85)		0.84 (0.78-0.91)		0.87 (0.79-0.95)
	Orkney		0.81 (0.63-1.06)		0.74 (0.60-0.90)		0.60 (0.35-1.02)		0.83 (0.43-1.60)
	Shetland		0.69 (0.51-0.95)		0.77 (0.72-0.82)		1.09 (0.69-1.71)		1.74 (0.93-3.25)
	Western Isles		0.69 (0.58-0.84)		0.59 (0.78-0.88)		0.51 (0.36-0.73)		0.69 (0.44-1.09)
	Fife		0.77 (0.73-0.81)		0.77 (0.93-1.01)		0.74 (0.68-0.82)		0.89 (0.78-1.00)
	Tayside		0.84 (0.79-0.88)		0.83 (0.96-1.06)		0.88 (0.80-0.96)		0.94 (0.84-1.05)
	Greater Glasgow and Clyde		0.95 (0.91-0.99)		0.97 (0.83-1.00)		0.96 (0.90-1.03)		1.03 (0.94-1.11)
	Lanarkshire		0.99 (0.95-1.03)		1.01 (0.87-1.03)		0.99 (0.92-1.06)		1.03 (0.94-1.12)

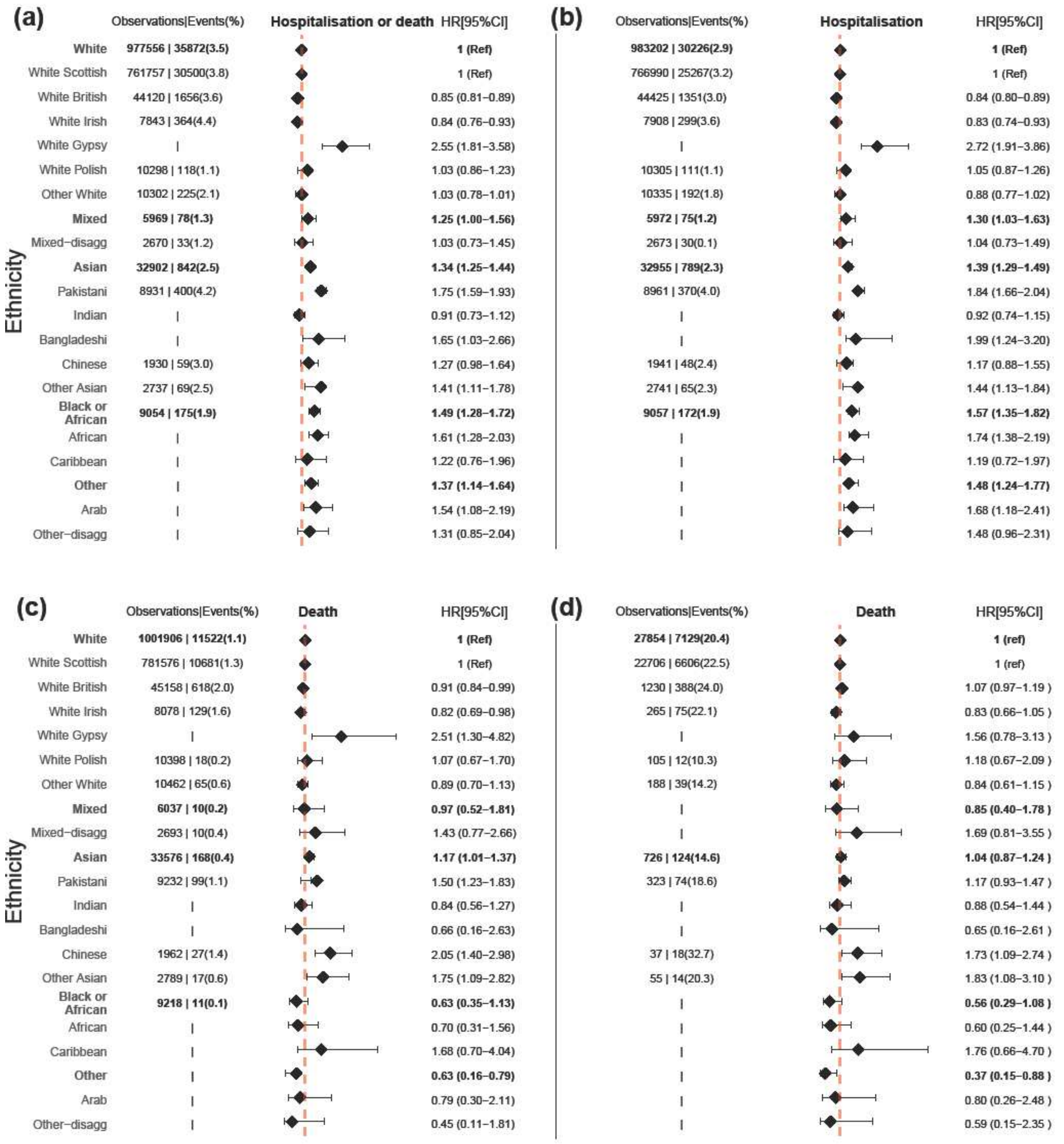

Figure S2: Ethnic differences in the risk of COVID-19 related outcomes: (a) hospitalisations or deaths following confirmed positive SARS-CoV-2 test, (b) hospitalisations following confirmed positive SARS-CoV-2 test, (c) deaths following confirmed positive SARS-CoV-2 test, and (d) deaths following COVID-19 hospitalisation

Models adjusted for age, sex and health board. Bold estimates are for aggregated ethic groups with White group as the reference category. The non-bold estimates are for disaggregated ethnic groups with White Scottish as the reference category. Data are observations and events, HR (95%CI) = Hazard ratio (95% Confidence Interval. The bold and non-bold HR estimates are not comparable since they are based on different reference categories. Observations and Events for ethnic groups containing small numbers (≤10) were removed as per data disclosure agreements and indicated by only low numbers not allowable for disclosure release are represented by |.

Amele S, *et al. J Epidemiol Community Health* 2023; 77:641–648. doi: 10.1136/jech-2023-220501

Table S7: Ethnic differences in the risk of COVID-19 related hospitalisations and deaths, hospitalisations and deaths based on 5 aggregated ethnic categories across waves following a confirmed positive SARS-CoV-2 test and COVID-19 hospitalisation where primay position of hospitalisation or death was only COVID-19.

**Variable**	**Categories**	**Confirmed positive SARS-CoV-2 test**						**COVID-19 hospitalisation**	
		**Hospitalisations and deaths**		**Hospitalisations**		**Deaths**		**Deaths**	
		Age-sex adjusted	Plus health board	Age-sex adjusted	Plus health board	Age-sex adjusted	Plus health board	Age-sex adjusted	Plus health board
		HR (95% CI)	HR (95% CI)	HR (95% CI)	HR (95% CI)	HR (95% CI)	HR (95% CI)	HR (95% CI)	HR (95% CI)
Ethnic group	White	1(ref)	1(ref)	1(ref)	1(ref)	1(ref)	1(ref)	1(ref)	1(ref)
	Mixed	1.49 (1.13-1.95)	1.51 (1.15-1.98)	1.56 (1.18-2.07)	1.60 (1.21-2.12)	1.20 (0.63-2.32)	1.20 (0.62-2.30)	1.20 (0.54-2.67)	1.20 (0.54-2.67)
	Asian	1.65 (1.52-1.79)	1.62 (1.49-1.75)	1.72 (1.58-1.88)	1.69 (1.55-1.84)	1.42 (1.21-1.67)	1.36 (1.15-1.60)	1.31 (1.07-1.61)	1.26 (1.03-1.55)
	Black	1.72 (1.43-2.06)	1.74 (1.45-2.09)	1.82 (1.51-2.19)	1.87 (1.55-2.25)	0.71 (0.37-1.36)	0.69 (0.36-1.33)	0.52 (0.22-1.25)	0.50 (0.21-1.21)
	Other	1.62 (1.31-2.01)	1.64 (1.32-2.03)	1.80 (1.45-2.23)	1.83 (1.47-2.27)	0.32 (0.12-0.86)	0.32 (0.12-0.85)	0.35 (0.11-1.09)	0.36 (0.12-1.11)
Age	16-20	Ref	Ref	Ref	Ref	Ref	Ref	Ref	Ref
	21-25	1.89 (1.46-2.44)	1.88 (1.45-2.44)	1.89 (1.46-2.45)	1.89 (1.45-2.44)	1.65 (0.15-18.21)	1.65 (0.15-18.15)	1.02 (0.00-Inf)	1.00 (0.00-Inf)
	26-30	2.94 (2.30-3.75)	2.93 (2.29-3.74)	2.93 (2.29-3.75)	2.92 (2.28-3.74)	5.96 (0.73-48.42)	5.92 (0.73-48.11)	131111.70 (0.00-Inf)	129991.53 (0.00-Inf)
	31-35	3.91 (3.08-4.96)	3.89 (3.07-4.94)	3.90 (3.07-4.96)	3.88 (3.05-4.94)	11.63 (1.52-88.94)	11.55 (1.51-88.31)	279955.40 (0.00-Inf)	276081.17 (0.00-Inf)
	36-40	5.49 (4.36-6.93)	5.47 (4.34-6.90)	5.46 (4.32-6.90)	5.44 (4.31-6.87)	21.03 (2.84-155.72)	20.90 (2.82-154.75)	401744.70 (0.00-Inf)	396478.71 (0.00-Inf)
	41-45	7.96 (6.33-10.01)	7.95 (6.32-9.99)	7.72 (6.13-9.73)	7.72 (6.13-9.72)	54.75 (7.57-396.06)	54.42 (7.54-394.36)	761759.80 (0.00-Inf)	749359.01 (0.00-Inf)
	46-50	11.52 (9.20-14.43)	11.44 (9.14-14.33)	11.24 (8.96-14.10)	11.15 (8.89-13.99)	97.21 (13.55-697.55)	96.54 (13.45-692.75)	89877.70 (0.00-Inf)	1071753.10 (0.00-Inf)
	51-55	18.44 (14.78-23.01)	18.22 (14.60-22.74)	17.94 (14.35-22.42)	17.69 (14.16-22.11)	178.14 (24.95-1272.11)	176.17 (24.67-1258.10)	1390033.00 (0.00-Inf)	1355587.03 (0.00-Inf)
	56-60	28.82 (23.12-35.91)	28.38 (22.77-35.37)	27.28 (21.86-34.05)	26.80 (21.47-33.45)	396.16 (55.63-2821.33)	390.65 (54.85-2782.10)	1934190.26 (0.00-Inf)	1887521.68 (0.00-Inf)
	61-65	46.9 (37.68-58.47)	46.25 (37.13-57.61)	43.38 (34.76-54.13)	42.61 (34.15-53.17)	898.01 (126.23-6388.33)	886.68 (124.64-6307.69)	296240.13 (0.00-Inf)	2897352.21 (0.00-Inf)
	66-70	84.17 (67.58-104.83)	83.21 (66.81-103.64)	75.76 (60.72-94.53)	74.65 (59.83-93.15)	1922.76 (270.40-13672.35)	1907.84 (268.30-13566.22)	3656738.02 (0.00-Inf)	3588705.26 (0.00-Inf)
	71-75	151.05 (121.42-187.92)	149.50 (120.17-185.98)	127.47 (102.27-158.87)	125.81 (100.94-156.81)	4489.36 (631.81-31899.46)	4452.61 (626.64-31638.38)	5154736.02 (0.00-Inf)	5060399.86 (0.00-Inf)
	76-80	237.35 (190.82-295.24)	233.93 (188.07-290.99)	183.01 (146.81-228.13)	179.81 (144.25-224.15)	7824.59 (1101.34-55590.83)	7732.52 (1088.38-54936.77)	5267020.80 (0.00-Inf)	5162892.67 (0.00-Inf)
	81-85	338.32 (272.06-420.71)	333.98 (268.57-415.32)	229.68 (184.24-286.32)	226.02 (181.31-281.77)	13679.89 (1925.86-97171.57)	13527.77 (1904.45-96091.19)	6389295.91 (0.00-Inf)	6245274.54 (0.00-Inf)
	86-90	398.93 (320.57-496.44)	396.84 (318.89-493.84)	224.55 (179.77-280.48)	223.02 (178.55-278.57)	19889.86 (2799.99-141288.50)	19793.57 (2786.44-140604.51)	7333054.31 (0.00-Inf)	7205491.22 (0.00-Inf)
	>90	440.57 (353.25-549.47)	440.60 (353.28-549.51)	172.59 (137.16-217.19)	172.51 (137.09-217.08)	28542.57 (4017.17-202799.19)	28552.92 (4018.62-202872.84)	8572249.02 (0.00-Inf)	8397190.68 (0.00-Inf)
Sex	Male	Ref	Ref	Ref	Ref	Ref	Ref	Ref	Ref
	Female	0.76 (0.74-0.78)	0.76 (0.74-0.78)	0.78 (0.76-0.81)	0.78 (0.76-0.80)	0.62 (0.60-0.65)	0.62 (0.60-0.65)	0.7 (0.66-0.74)	0.69 (0.65-0.74)
Health Board	Ayrshire and Arran		Ref		Ref		Ref		Ref
	Borders		0.85 (0.76-0.94)		0.91 (0.81-1.02)		0.81 (0.68-0.96)		1.1 (0.89-1.36)
	Dumfries and Galloway		0.91 (0.83-1.00)		0.98 (0.89-1.08)		0.67 (0.57-0.79)		0.80 (0.64-1.00)
	Forth Valley		0.99 (0.93-1.06)		1.02 (0.94-1.09)		0.90 (0.80-1.00)		0.95 (0.82-1.10)
	Grampian		0.73 (0.69-0.78)		0.73 (0.67-0.78)		0.71 (0.64-0.79)		0.86 (0.74-1.01)
	Highland		0.66 (0.61-0.72)		0.63 (0.57-0.69)		0.65 (0.56-0.74)		0.69 (0.56-0.86)

	Lothian		0.74 (0.70-0.79)		0.68 (0.64-0.72)		0.86 (0.78-0.93)		0.84 (0.74-0.95)
	Orkney		0.65 (0.45-0.93)		0.75 (0.52-1.09)		0.35 (0.16-0.79)		0.64 (0.24-1.71)
	Shetland		0.76 (0.53-1.10)		0.57 (0.36-0.91)		1.17 (0.70-1.95)		1.40 (0.58-3.38)
	Western Isles		0.60 (0.47-0.77)		0.67 (0.51-0.87)		0.39 (0.24-0.62)		0.58 (0.31-1.09)
	Fife		0.74 (0.69-0.79)		0.71 (0.66-0.77)		0.75 (0.67-0.84)		0.87 (0.75-1.02)
	Tayside		0.92 (0.87-0.98)		0.93 (0.86-0.99)		0.91 (0.82-1.00)		1.00 (0.87-1.15)
	Greater Glasgow and Clyde		0.98 (0.93-1.03)		0.98 (0.93-1.04)		0.98 (0.91-1.06)		1.05 (0.94-1.16)
	Lanarkshire		0.99 (0.94-1.04)		0.99 (0.93-1.05)		1.00 (0.92-1.09)		1.02 (0.91-1.15)

Table S8: Ethnic differences in the risk of COVID-19 related hospitalisations and deaths, hospitalisations and deaths based on 16 disaggregated ethnic subcategories across waves following a confirmed positive SARS-CoV-2 test and COVID-19 hospitalisation where primay position of hospitalisation or death was only COVID-19.

Variable	Categories	Confirmed positive SARS-CoV-2 test						COVID-19 hospitalisation	
		Hospitalisations and deaths		Hospitalisations		Deaths		Deaths	
		Age-sex adjusted	Plus health board	Age-sex adjusted	Plus health board	Age-sex adjusted	Plus health board	Age-sex adjusted	Plus health board
		HR (95% CI)	HR (95% CI)	HR (95% CI)	HR (95% CI)	HR (95% CI)	HR (95% CI)	HR (95% CI)	HR (95% CI)
Ethnic group	White Scottish	1(ref)	1(ref)	1(ref)	1(ref)	1(ref)	1(ref)	1(ref)	1(ref)
	White Other British	0.79 (0.74-0.84)	0.83 (0.78-0.88)	0.77 (0.71-0.82)	0.81 (0.75-0.87)	0.85 (0.77-0.93)	0.91 (0.82-1.00)	0.97 (0.85-1.11)	1.02 (0.89-1.16)
	White Irish	0.86 (0.76-0.98)	0.83 (0.73-0.94)	0.85 (0.73-0.98)	0.82 (0.71-0.95)	0.81 (0.66-1.00)	0.77 (0.63-0.95)	0.77 (0.56-1.04)	0.74 (0.55-1.01)
	White Gypsy/Traveller	2.47 (1.59-3.83)	2.46 (1.59-3.82)	2.90 (1.87-4.50)	2.90 (1.87-4.49)	1.87 (0.78-4.48)	1.85 (0.77-4.45)	1.47 (0.61-3.55)	1.50 (0.62-3.60)
	White Polish	1.02 (0.81-1.29)	1.09 (0.86-1.37)	1.03 (0.81-1.32)	1.11 (0.87-1.42)	1.01 (0.58-1.74)	1.05 (0.61-1.81)	1.20 (0.60-2.41)	1.25 (0.62-2.51)
	Other White	0.94 (0.80-1.10)	0.97 (0.83-1.14)	0.95 (0.80-1.13)	1.00 (0.84-1.19)	0.83 (0.62-1.10)	0.84 (0.63-1.12)	0.80 (0.53-1.20)	0.80 (0.53-1.21)
	Mixed - disaggregate	1.17 (0.77-1.78)	1.20 (0.79-1.82)	1.17 (0.75-1.84)	1.21 (0.77-1.90)	1.73 (0.90-3.32)	1.74 (0.90-3.34)	2.36 (1.06-5.26)	2.39 (1.07-5.32)
	Pakistani	2.15 (1.92-2.42)	2.08 (1.85-2.33)	2.28 (2.01-2.57)	2.20 (1.94-2.49)	1.77 (1.43-2.20)	1.69 (1.36-2.09)	1.40 (1.07-1.83)	1.34 (1.02-1.76)
	Indian	0.97 (0.75-1.26)	0.95 (0.74-1.24)	1.04 (0.79-1.36)	1.02 (0.78-1.34)	0.91 (0.58-1.45)	0.87 (0.55-1.38)	1.20 (0.70-2.08)	1.13 (0.66-1.95)
	Bangladeshi	2.09 (1.24-3.54)	2.21 (1.31-3.73)	2.57 (1.52-4.34)	2.82 (1.67-4.77)	0.87 (0.22-3.49)	0.86 (0.22-3.46)	1.04 (0.26-4.15)	1.07 (0.27-4.30)
	Chinese	1.61 (1.21-2.15)	1.59 (1.19-2.13)	1.41 (1.01-1.98)	1.41 (1.00-1.97)	2.82 (1.93-4.11)	2.72 (1.86-3.97)	2.36 (1.42-3.93)	2.26 (1.36-3.76)
	Other Asian	1.72 (1.31-2.25)	1.75 (1.34-2.30)	1.76 (1.33-2.34)	1.82 (1.37-2.42)	2.09 (1.26-3.47)	2.08 (1.25-3.45)	2.29 (1.27-4.14)	2.23 (1.23-4.03)
	African	1.84 (1.39-2.43)	1.87 (1.41-2.47)	1.94 (1.46-2.59)	1.99 (1.49-2.65)	0.96 (0.43-2.14)	0.94 (0.42-2.10)	0.59 (0.19-1.83)	0.57 (0.18-1.76)
	Caribbean	1.28 (0.71-2.31)	1.30 (0.72-2.34)	1.33 (0.72-2.48)	1.37 (0.73-2.54)	1.35 (0.44-4.19)	1.35 (0.43-4.18)	1.48 (0.37-5.91)	1.47 (0.37-5.90)
	Arab	1.86 (1.24-2.81)	1.88 (1.25-2.83)	2.09 (1.37-3.17)	2.12 (1.40-3.23)	1.06 (0.40-2.84)	1.06 (0.40-2.81)	1.25 (0.40-3.87)	1.28 (0.41-3.97)
	Other Ethnic Group	1.27 (0.72-2.24)	1.29 (0.73-2.26)	1.50 (0.85-2.65)	1.53 (0.87-2.70)	0 .00 (0-Inf)	0.00 (0.00-Inf)	0.00 (0.00-Inf)	0.00 (0.00-Inf)
Age	16-20	Ref	Ref	Ref	Ref	Ref	Ref	Ref	Ref
	21-25	1.94 (1.45-2.60)	1.94 (1.45-2.59)	1.95 (1.45-2.61)	1.94 (1.45-2.60)	1.68 (0.15-18.53)	1.67 (0.15-18.45)	1.02 (0.00-Inf)	1.01 (0.00-Inf)
	26-30	3.17 (2.40-4.18)	3.14 (2.38-4.15)	3.16 (2.39-4.18)	3.14 (2.37-4.14)	6.57 (0.81-53.42)	6.51 (0.80-52.94)	149994.00 (0-Inf)	149437.05 (0-Inf)
	31-35	4.24 (3.24-5.57)	4.21 (3.21-5.52)	4.23 (3.22-5.56)	4.19 (3.19-5.51)	13.45 (1.76-102.81)	13.32 (1.74-101.81)	337805.97 (0-Inf)	334035.76 (0-Inf)
	36-40	5.55 (4.26-7.23)	5.51 (4.23-7.18)	5.50 (4.21-7.18)	5.46 (4.18-7.12)	23.49 (3.17-173.94)	23.28 (3.14-172.41)	488115.12 (0-Inf)	482732.32 (0-Inf)
	41-45	7.79 (6.01-10.11)	7.76 (5.99-10.06)	7.47 (5.74-9.71)	7.44 (5.72-9.67)	57.74 (7.98-417.64)	57.34 (7.93-414.81)	913433.84 (0-Inf)	905207.82 (0-Inf)
	46-50	11.66 (9.04-15.03)	11.57 (8.98-14.92)	11.29 (8.74-14.59)	11.20 (8.67-14.47)	97.27 (13.56-698.02)	96.44 (13.44-692.02)	1191579.79 (0-Inf)	1173884.49 (0-Inf)
	51-55	19.32 (15.05-24.80)	19.11 (14.89-24.53)	18.70 (14.53-24.05)	18.47 (14.36-23.76)	173.58 (24.31-1239.58)	171.56 (24.02-1225.13)	1446356.85 (0-Inf)	1416177.14 (0-Inf)
	56-60	30.32 (23.66-38.85)	29.91 (23.34-38.33)	28.46 (22.16-36.56)	28.03 (21.82-36.00)	379.49 (53.29-2702.58)	373.93 (52.51-2663.00)	1952828.53 (0-Inf)	1911372.96 (0-Inf)
	61-65	50.00 (39.03-	49.34 (38.52-63.20)	45.77 (35.65-	45.07 (35.11-57.87)	850.49 (119.56-	838.87 (117.92-	2896130.02 (0-Inf)	2842894.51 (0-Inf)

		64.04)		58.76)		6050.25)	5967.55)		
	66-70	90.66 (70.80-116.10)	89.74 (70.08-114.91)	80.91 (63.04-103.86)	79.88 (62.23-102.54)	1789.59 (251.67-12725.36)	1773.10 (249.35-12608.06)	3486546.42 (0-Inf)	3431533.76 (0-Inf)
	71-75	166.54 (130.21-213.00)	164.97 (128.98-211.00)	139.18 (108.55-178.45)	137.61 (107.33-176.44)	4167.17 (586.47-29610.06)	4126.83 (580.79-29323.46)	4816542.84 (0-Inf)	4743631.69 (0-Inf)
	76-80	261.86 (204.77-334.86)	258.60 (202.22-330.70)	199.57 (155.64-255.90)	196.65 (153.36-252.15)	7159.46 (1007.72-50865.14)	7069.11 (995.00-50223.30)	4831697.17 (0-Inf)	4751577.27 (0-Inf)
	81-85	375.26 (293.53-479.76)	371.30 (290.43-474.69)	250.90 (195.67-321.72)	247.71 (193.18-317.64)	12480.49 (1757.02-88651.68)	12334.43 (1736.45-87614.38)	5834200.21 (0-Inf)	5721524.85 (0-Inf)
	86-90	441.76 (345.32-565.13)	439.84 (343.82-562.68)	244.12 (190.04-313.59)	242.87 (189.07-311.99)	18012.69 (2535.74-127953.43)	17900.17 (2519.90-127154.31)	6656290.85 (0-Inf)	6560586.26 (0-Inf)
	>90	492.20 (383.99-630.89)	492.32 (384.09-631.05)	188.55 (145.78-243.86)	188.65 (145.86-243.99)	25829.00 (3635.26-183518.27)	25785.69 (3629.16-183210.90)	7658489.57 (0-Inf)	7534040.23 (0-Inf)
sex	Male	Ref	Ref	Ref	Ref	Ref	Ref	Ref	Ref
	Female	0.76 (0.74-0.78)	0.76 (0.74-0.78)	0.78 (0.76-0.81)	0.78 (0.76-0.80)	0.63 (0.60-0.65)	0.62 (0.60-0.65)	0.69 (0.65-0.74)	0.60 (0.65-0.73)
Health Board	Ayrshire and Arran		Ref		Ref		Ref		Ref
	Borders		0.87 (0.78-0.97)		0.95 (0.84-1.08)		0.80 (0.67-0.95)		1.05 (0.85-1.30)
	Dumfries and Galloway		0.93 (0.85-1.02)		1.01 (0.91-1.12)		0.69 (0.59-0.81)		0.81 (0.65-1.01)
	Forth Valley		0.99 (0.93-1.06)		1.03 (0.95-1.11)		0.88 (0.79-0.99)		0.94 (0.81-1.09)
	Grampian		0.73 (0.69-0.78)		0.73 (0.67-0.79)		0.70 (0.63-0.78)		0.86 (0.74-1.01)
	Highland		0.67 (0.62-0.73)		0.63 (0.57-0.70)		0.66 (0.58-0.75)		0.71 (0.57-0.88)
	Lothian		0.77 (0.72-0.81)		0.70 (0.65-0.75)		0.86 (0.79-0.94)		0.84 (0.74-0.95)
	Orkney		0.65 (0.45-0.95)		0.76 (0.52-1.13)		0.35 (0.16-0.78)		0.60 (0.22-1.60)
	Shetland		0.79 (0.55-1.15)		0.60 (0.37-0.96)		1.15 (0.69-1.92)		1.40 (0.58-3.38)
	Western Isles		0.57 (0.44-0.74)		0.63 (0.47-0.84)		0.40 (0.25-0.64)		0.61 (0.33-1.15)
	Fife		0.74 (0.69-0.80)		0.71 (0.66-0.77)		0.75 (0.67-0.84)		0.87 (0.74-1.01)
	Tayside		0.93 (0.87-0.99)		0.93 (0.87-1.01)		0.90 (0.82-1.00)		1.00 (0.87-1.15)
	Greater Glasgow and Clyde		0.98 (0.93-1.03)		0.98 (0.93-1.04)		0.99 (0.92-1.07)		1.07 (0.96-1.19)
	Lanarkshire		0.99 (0.93-1.04)		0.99 (0.93-1.05)		0.99 (0.91-1.08)		1.02 (0.91-1.15)

Table S9: Ethnic differences in the risk of COVID-19 related hospitalisations and deaths, hospitalisations, deaths and positive SARS-CoV-2 tests based on aggregated 5 ethnic categories by waves.

Ethnic group	Age-sex adjusted	Plus health board	Age-sex adjusted	Plus health board	Age-sex adjusted	Plus health board	Age-sex adjusted	Plus health board
	HR (95% CI)	HR (95% CI)	HR (95% CI)	HR (95% CI)	HR (95% CI)	HR (95% CI)	HR (95% CI)	HR (95% CI)
**COVID-19 hospitalisation or death**								
	**Wave 1**		**Wave 2**		**Wave 3**		**Wave 4**	
White	1 (ref)	1 (ref)	1 (ref)	1 (ref)	1(ref)	1(ref)	1(ref)	1(ref)
Mixed or multiple ethnic groups	1.47 (0.83-2.59)	1.40 (0.79-2.47)	0.99 (0.66-1.50)	0.99 (0.66-1.49)	1.62 (1.19-2.21)	1.63 (1.19-2.22)	0.57 (0.34-0.94)	0.57 (0.34-0.95)
Asian	1.35 (1.12-1.64)	1.14 (0.94-1.38)	1.82 (1.64-2.02)	1.63 (1.47-1.81)	1.40 (1.24-1.58)	1.34 (1.18-1.51)	0.86 (0.74-1.00)	0.83 (0.71-0.96)

Black	1.58 (1.03-2.42)	1.44 (0.93-2.21)	1.77 (1.40-2.25)	1.75 (1.38-2.21)	1.62 (1.27-2.06)	1.63 (1.28-2.07)	0.68 (0.47-0.98)	0.69 (0.48-0.99)
Other ethnic group	0.56 (0.25-1.24)	0.52 (0.23-1.16)	1.31 (0.96-1.79)	1.31 (0.96-1.78)	1.67 (1.27-2.19)	1.67 (1.27-2.20)	1.06 (0.76-1.48)	1.07 (0.77-1.49)
**COVID-19 hospitalisation**								
	Wave 1		Wave 2		Wave 3		Wave 4	
White	1(ref)	1(ref)	1(ref)	1(ref)	1(ref)	1(ref)	1(ref)	1(ref)
Mixed or multiple ethnic groups	1.55 (0.83-2.88)	1.49 (0.80-2.78)	1.07 (0.71-1.62)	0.86 (0.80-0.91)	1.64 (1.20-2.25)	1.64 (1.20-2.25)	0.55 (0.33-0.93)	0.55 (0.33-0.93)
Asian	1.51 (1.22-1.86)	1.30 (1.05-1.61)	1.88 (1.69-2.10)	1.43 (1.40-1.46)	1.39 (1.23-1.58)	1.33 (1.17-1.51)	0.87 (0.75-1.02)	0.83 (0.72-0.97)
Black	1.91 (1.24-2.94)	1.78 (1.16-2.74)	1.87 (1.47-2.37)	1.27 (1.21-1.33)	1.65 (1.29-2.11)	1.66 (1.30-2.12)	0.68 (0.47-0.99)	0.68 (0.47-0.99)
Other ethnic group	0.70 (0.31-1.55)	0.66 (0.30-1.48)	1.42 (1.04-1.93)	1.21 (1.15-1.28)	1.70 (1.29-2.24)	1.70 (1.29-2.25)	1.01 (0.71-1.43)	1.02 (0.72-1.44)
**COVID-19 death**								
	Wave 1		Wave 2		Wave 3		Wave 4	
White	1(ref)	1(ref)	1(ref)	1(ref)	1(ref)	1(ref)	1(ref)	1(ref)
Mixed or multiple ethnic groups	1.75 (0.73-4.20)	1.66 (0.69-3.99)	0.64 (0.20-1.97)	0.63 (0.20-1.96)	0.81 (0.20-3.26)	0.82 (0.20-3.28)	0.47 (0.07-3.30)	0.47 (0.07-3.34)
Asian	1.34 (1.00-1.79)	1.08 (0.81-1.45)	1.51 (1.21-1.88)	1.31 (1.05-1.63)	1.59 (1.16-2.18)	1.51 (1.10-2.07)	0.83 (0.53-1.29)	0.79 (0.51-1.23)
Black	0.00 (0.00-Inf)	0.00 (0.00-Inf)	1.00 (0.47-2.10)	0.96 (0.46-2.02)	0.50 (0.13-2.01)	0.50 (0.13-2.02)	0.62 (0.16-2.49)	0.63 (0.16-2.54)
Other ethnic group	0.00 (0.00-Inf)	0.00 (0.00-Inf)	0.32 (0.08-1.27)	0.31 (0.08-1.24)	0.59 (0.15-2.37)	0.59 (0.15-2.38)	1.41 (0.53-3.76)	1.42 (0.53-3.80)
**Positive SARS-CoV-2 test**								
	Wave 1		Wave 2		Wave 3		Wave 4	
White	1(ref)	1(ref)	1(ref)	1(ref)	1(ref)	1(ref)	1(ref)	1(ref)
Mixed or multiple ethnic groups	0.89 (0.68-1.16)	0.86 (0.66-1.12)	0.87 (0.81-0.92)	0.86 (0.80-0.91)	0.80 (0.77-0.83)	0.79 (0.75-0.82)	0.80 (0.78-0.83)	0.80 (0.77-0.83)
Asian	1.77 (1.65-1.91)	1.64 (1.52-1.77)	1.53 (1.50-1.56)	1.43 (1.40-1.46)	0.69 (0.68-0.71)	0.67 (0.66-0.68)	0.64 (0.63-0.65)	0.63 (0.62-0.64)
Black	2.10 (1.82-2.41)	2.00 (1.74-2.30)	1.31 (1.25-1.37)	1.27 (1.21-1.33)	0.87 (0.84-0.90)	0.86 (0.83-0.89)	0.80 (0.78-0.83)	0.80 (0.77-0.82)
Other ethnic group	1.11 (0.89-1.39)	1.06 (0.85-1.33)	1.23 (1.17-1.30)	1.21 (1.15-1.28)	0.79 (0.76-0.82)	0.78 (0.75-0.81)	0.62 (0.60-0.65)	0.62 (0.60-0.65)

HR (95%CI) = Hazard ratio (95% Confidence Interval)- wave 1: March 1, 2020 to July 31, 2020- wave 2: August 1, 2020 to April 30, 2021- wave 3: May 1, 2021 to December 17, 2021- wave 4 : December 18, 2021 to April 17, 2022

Table S10: Ethnic differences in the risk of COVID-19 related hospitalisations and deaths, hospitalisations, deaths and positive SARS-CoV-2 tests based on disaggregated ethnic subcategories by waves.

**Ethnic group**	**Age-sex adjusted**	**Plus health board**	**Age-sex adjusted**	**Plus health board**	**Age-sex adjusted**	**Plus health board**	**Age-sex adjusted**	**Plus health board**
	HR (95% CI)	HR (95% CI)	HR (95% CI)	HR (95% CI)	HR (95% CI)	HR (95% CI)	HR (95% CI)	HR (95% CI)
**COVID-19 hospitalisation or death**								
	**Wave 1**		**Wave 2**		**Wave 3**		**Wave 4**	
White Scottish	1(ref)	1(ref)	1(ref)	1(ref)	1(ref)	1(ref)	1(ref)	1(ref)
White Other British	0.60 (0.53-0.67)	0.73 (0.65-0.81)	0.51 (0.47-0.55)	0.65 (0.59-0.70)	0.55 (0.50-0.61)	0.61 (0.55-0.67)	0.69 (0.64-0.74)	0.73 (0.68-0.80)
White Irish	1.13 (0.92-1.38)	0.98 (0.79-1.20)	0.97 (0.83-1.13)	0.85 (0.73-1.00)	0.82 (0.66-1.01)	0.78 (0.63-0.97)	0.83 (0.70-1.00)	0.77 (0.63-0.93)
White Gypsy/Traveller	0.82 (0.20-3.27)	0.88 (0.22-3.53)	1.73 (0.93-3.22)	1.91 (1.03-3.55)	1.78 (0.89-3.55)	1.85 (0.92-3.70)	3.23 (2.03-5.12)	.82 (2.41-6.07)
White Polish	0.52 (0.26-1.03)	0.54 (0.27-1.07)	0.66 (0.46-0.95)	0.75 (0.52-1.09)	1.23 (0.94-1.61)	1.33 (1.02-1.74)	0.82 (0.60-1.11)	0.87 (0.62-1.20)
Other White	0.71 (0.52-0.96)	0.73 (0.54-0.99)	0.66 (0.54-0.82)	0.74 (0.60-0.91)	0.66 (0.52-0.84)	0.70 (0.55-0.89)	0.61 (0.48-0.76)	0.63 (0.50-0.80)
Mixed or multiple ethnic groups	1.32 (0.63-2.77)	1.34 (0.64-2.81)	0.66 (0.34-1.26)	0.70 (0.37-1.35)	1.19 (0.72-1.97)	1.23 (0.74-2.05)	0.33 (0.14-0.78)	0.30 (0.11-0.81)
Pakistani	1.87 (1.40-2.50)	1.49 (1.12-2.00)	3.03 (2.62-3.52)	2.57 (2.22-2.99)	2.92 (2.47-3.45)	2.72 (2.30-3.22)	1.43 (1.15-1.77)	1.30 (1.04-1.64)
Indian	1.05 (0.64-1.72)	0.89 (0.54-1.45)	1.07 (0.77-1.47)	0.96 (0.70-1.32)	0.77 (0.51-1.17)	0.74 (0.49-1.13)	0.70 (0.47-1.04)	0.67 (0.44-1.02)
Bangladeshi	0.65 (0.09-4.65)	0.66 (0.09-4.71)	2.73 (1.47-5.07)	3.19 (1.72-5.93)	1.59 (0.66-3.81)	1.75 (0.73-4.20)	0.78 (0.25-2.43)	0.97 (0.31-3.00)
Chinese	0.62 (0.32-1.20)	0.56 (0.29-1.08)	0.72 (0.48-1.07)	0.69 (0.46-1.03)	0.61 (0.37-1.00)	0.61 (0.37-0.99)	0.66 (0.44-1.01)	0.64 (0.41-1.01)
Other Asian	3.24 (2.15-4.89)	3.12 (2.07-4.71)	1.29 (0.87-1.93)	1.35 (0.90-2.02)	0.67 (0.37-1.22)	0.70 (0.39-1.26)	0.98 (0.63-1.54)	0.96 (0.59-1.57)
African	2.00 (1.13-3.53)	1.82 (1.03-3.21)	1.67 (1.15-2.43)	1.67 (1.15-2.42)	1.92 (1.35-2.73)	1.96 (1.37-2.79)	0.38 (0.18-0.80)	0.43 (0.21-0.91)
Caribbean or Black	0.74 (0.18-2.95)	0.74 (0.18-2.95)	1.24 (0.62-2.47)	1.32 (0.66-2.63)	0.97 (0.40-2.33)	1.00 (0.42-2.41)	0.47 (0.15-1.46)	0.55 (0.18-1.71)
Arab	0.33 (0.05-2.31)	0.30 (0.04-2.16)	1.66 (0.97-2.87)	1.65 (0.96-2.84)	1.80 (1.02-3.17)	1.80 (1.02-3.18)	1.12 (0.58-2.16)	1.13 (0.57-2.26)
Other Ethnic Group	0.39 (0.05-2.75)	0.36 (0.05-2.57)	1.25 (0.63-2.51)	1.26 (0.63-2.51)	1.94 (1.05-3.62)	1.97 (1.06-3.66)	0.48 (0.15-1.48)	0.54 (0.18-1.69)

**COVID-19 hospitalisation**								
	**Wave 1**		**Wave 2**		**Wave 3**		**Wave 4**	
White Scottish	1(ref)	1(ref)	1(ref)	1(ref)	1(ref)	1(ref)	1(ref)	1(ref)
White Other British	0.59 (0.51-0.68)	0.72 (0.62-0.82)	0.51 (0.46-0.55)	0.65 (0.59-0.71)	0.54 (0.49-0.60)	0.61 (0.55-0.67)	0.67 (0.62-0.73)	0.75 (0.69-0.82)
White Irish	1.12 (0.86-1.46)	0.98 (0.75-1.28)	0.98 (0.83-1.17)	0.86 (0.73-1.03)	0.81 (0.64-1.01)	0.77 (0.62-0.97)	0.81 (0.66-1.00)	0.76 (0.62-0.94)
White Gypsy/Traveller	1.13 (0.28-4.51)	1.25 (0.31-5.00)	1.95 (1.05-3.63)	2.17 (1.17-4.03)	1.88 (0.94-3.76)	1.96 (0.98-3.91)	3.71 (2.30-5.97)	3.92 (2.44-6.31)
White Polish	0.58 (0.28-1.23)	0.62 (0.30-1.31)	0.72 (0.50-1.04)	0.84 (0.58-1.21)	1.10 (0.82-1.47)	1.19 (0.89-1.58)	0.85 (0.61-1.17)	0.91 (0.66-1.27)
Other White	0.74 (0.51-1.06)	0.78 (0.54-1.13)	1.95 (0.55-0.86)	0.78 (0.62-0.97)	0.63 (0.49-0.81)	0.67 (0.52-0.86)	0.59 (0.46-0.76)	0.62 (0.48-0.80)
Mixed or multiple ethnic groups	1.26 (0.52-3.02)	1.3 (0.54-3.12)	0.72 (0.38-1.40)	0.79 (0.41-1.51)	1.16 (0.69-1.96)	1.20 (0.71-2.04)	0.23 (0.08-0.72)	0.24 (0.08-0.75)
Pakistani	2.11 (1.52-2.92)	1.72 (1.24-2.38)	0.69 (2.73-3.72)	2.69 (2.31-3.14)	2.93 (2.47-3.49)	2.74 (2.30-3.25)	1.35 (1.06-1.72)	1.25 (0.98-1.59)
Indian	1.13 (0.64-1.99)	0.98 (0.56-1.73)	0.73 (0.78-1.52)	0.98 (0.70-1.38)	0.74 (0.48-1.15)	0.72 (0.46-1.12)	0.76 (0.50-1.15)	0.73 (0.48-1.11)
Bangladeshi	0 (0,inf)	0 (0-inf)	3.19 (1.65-5.69)	3.71 (2.00-6.90)	1.67 (0.70-4.02)	1.85 (0.77-4.44)	0.95 (0.31-2.95)	1.05 (0.34-3.25)
Chinese	0.50 (0.21-1.21)	0.47 (0.20-1.13)	1.09 (0.42-1.02)	0.63 (0.40-0.99)	0.49 (0.28-0.86)	0.48 (0.27-0.85)	0.71 (0.45-1.11)	0.70 (0.45-1.10)
Other Asian	3.98 (2.61-6.06)	3.93 (2.58-5.99)	3.06 (0.81-1.90)	1.31 (0.85-2.01)	0.70 (0.39-1.27)	0.73 (0.40-1.32)	0.99 (0.60-1.61)	1.02 (0.63-1.67)
African	2.46 (1.39-4.33)	2.30 (1.30-4.05)	0.65 (1.25-2.62)	1.82 (1.25-2.64)	1.94 (1.35-2.77)	1.98 (1.38-2.83)	0.45 (0.21-0.94)	0.45 (0.22-0.95)
Caribbean	1.02 (0.26-4.10)	1.05 (0.26-4.20)	1.24 (0.58-2.56)	1.31 (0.63-2.75)	1.03 (0.43-2.47)	1.06 (0.44-2.55)	0.38 (0.10-1.53)	0.40 (0.10-1.58)
Arab	0.43 (0.06-3.04)	0.41 (0.06-2.91)	1.81 (1.07-3.18)	1.84 (1.07-3.17)	1.90 (1.08-3.34)	1.90 (1.08-3.35)	0.90 (0.41-2.01)	0.90 (0.41-2.01)
Other Ethnic Group	0.52 (0.07-3.69)	0.50 (0.07-3.52)	1.85 (0.70-2.80)	1.41 (0.71-2.83)	1.85 (0.96-3.56)	1.87 (0.97-3.60)	0.39 (0.10-1.55)	0.39 (0.10-1.56)
**COVID-19 death**								
	**Wave 1**		**Wave 2**		**Wave 3**		**Wave 4**	
White Scottish	1(ref)	1(ref)	1(ref)	1(ref)	1(ref)	1(ref)	1(ref)	1(ref)
White Other British	0.64 (0.55-0.73)	0.78 (0.68-0.90)	0.53 (0.47-0.60)	0.67 (0.59-0.76)	0.66 (0.55-0.79)	0.74 (0.62-0.89)	0.62 (0.52-0.74)	0.67 (0.56-0.81)
White Irish	0.99 (0.75-1.32)	0.85 (0.64-1.12)	0.89 (0.69-1.14)	0.77 (0.60-0.99)	0.66 (0.41-1.06)	0.62 (0.39-1.00)	1.00 (0.70-1.43)	0.94 (0.65-1.34)
White Gypsy/Traveller	0.00 (0.00-Inf)	0.00 (0.00-Inf)	1.10 (0.27-4.38)	1.18 (0.30-4.74)	3.73 (1.20-11.58)	3.87 (1.25-12.02)	5.36 (2.01-14.31)	5.49 (2.06-14.65)
White Polish	0.35 (0.09-1.39)	0.35 (0.09-1.41)	0.11 (0.02-0.77)	0.12 (0.02-0.85)	2.81 (1.65-4.79)	2.99 (1.75-5.09)	0.70 (0.23-2.19)	0.74 (0.24-2.30)

Other White	0.61 (0.38-0.97)	0.62 (0.39-0.98)	0.70 (0.49-0.99)	0.75 (0.52-1.06)	0.69 (0.40-1.19)	0.72 (0.42-1.25)	0.62 (0.35-1.09)	0.64 (0.37-1.14)
Mixed or multiple ethnic groups	2.23 (0.93-5.36)	2.25 (0.94-5.42)	0.82 (0.27-2.55)	0.88 (0.28-2.74)	1.15 (0.29-4.61)	1.19 (0.30-4.77)	0.63 (0.09-4.45)	0.65 (0.09-4.64)
Pakistani	1.95 (1.30-2.95)	1.52 (1.01-2.29)	2.45 (1.82-3.28)	2.04 (1.52-2.74)	3.31 (2.26-4.84)	3.06 (2.09-4.49)	1.77 (1.04-2.99)	1.66 (0.98-2.81)
Indian	0.93 (0.44-1.94)	0.75 (0.36-1.58)	1.11 (0.64-1.92)	0.96 (0.56-1.65)	0.97 (0.40-2.34)	0.92 (0.38-2.22)	0.19 (0.03-1.37)	0.18 (0.03-1.31)
Bangladeshi	1.41 (0.20-10.02)	1.34 (0.19-9.49)	0.91 (0.13-6.47)	0.97 (0.14-6.86)	0.00 (0.00-Inf)	0.00 (0.00-Inf)	2.05 (0.29-14.60)	2.15 (0.30-15.27)
Chinese	1.23 (0.64-2.37)	1.07 (0.56-2.07)	1.25 (0.74-2.12)	1.19 (0.70-2.00)	1.25 (0.56-2.78)	1.22 (0.55-2.73)	0.41 (0.10-1.65)	0.41 (0.10-1.64)
Other Asian	2.19 (0.98-4.88)	2.05 (0.92-4.57)	1.73 (0.87-3.47)	1.75 (0.87-3.50)	0.86 (0.22-3.46)	0.89 (0.22-3.56)	0.99 (0.25-3.97)	1.02 (0.25-4.08)
African	0.00 (0.00-Inf)	0.00 (0.00-Inf)	1.11 (0.41-2.95)	1.07 (0.40-2.85)	1.00 (0.25-4.02)	1.02 (0.25-4.08)	0.00 (0.00-0.00)	0.00 (0.00-0.00)
Caribbean	0.00 (0.00-Inf)	0.00 (0.00-Inf)	1.54 (0.50-4.77)	1.61 (0.52-5.01)	0.00 (0.00-Inf)	0.00 (0.00-Inf)	2.38 (0.59-9.52)	2.46 (0.62-9.85)
Arab	0.00 (0.00-Inf)	0.00 (0.00-Inf)	0.90 (0.22-3.59)	0.89 (0.22-3.55)	0.93 (0.13-6.59)	0.93 (0.13-6.60)	2.11 (0.53-8.43)	2.13 (0.53-8.53)
Other Ethnic Group	0.00 (0.00-Inf)	0.00 (0.00-Inf)	0 (0,inf)	0 (0,inf)	1.12 (0.16-7.93)	1.13 (0.16-8.02)	2.46 (0.61-9.83)	2.49 (0.62-9.98)
**Positive SARS-CoV-2 test**								
	**Wave 1**		**Wave 2**		**Wave 3**		**Wave 4**	
White Scottish	1(ref)	1(ref)	1(ref)	1(ref)	1(ref)	1(ref)	1(ref)	1(ref)
White Other British	0.71 (0.66-0.76)	0.80 (0.74-0.86)	0.56 (0.55-0.58)	0.67 (0.66-0.69)	0.71 (0.70-0.72)	0.76 (0.74-0.77)	0.77 (0.76-0.78)	0.83 (0.82-0.84)
White Irish	1.15 (1.00-1.33)	1.08 (0.93-1.25)	0.95 (0.90-1.01)	0.89 (0.84-0.94)	1.07 (1.03-1.11)	1.04 (1.00-1.07)	1.00 (0.97-1.03)	0.97 (0.94-1.00)
White Gypsy/Traveller	0.34 (0.11-1.07)	0.35 (0.11-1.09)	1.15 (0.96-1.37)	1.25 (1.05-1.49)	0.74 (0.65-0.85)	0.76 (0.66-0.87)	0.68 (0.60-0.76)	0.70 (0.62-0.79)
White Polish	0.91 (0.74-1.10)	0.93 (0.76-1.13)	1.06 (1.01-1.11)	1.19 (1.13-1.25)	0.84 (0.82-0.87)	0.86 (0.84-0.89)	0.97 (0.94-0.99)	1.00 (0.98-1.03)
Other White	0.73 (0.62-0.86)	0.75 (0.64-0.89)	0.62 (0.58-0.65)	0.68 (0.65-0.72)	0.70 (0.68-0.72)	0.72 (0.69-0.74)	0.72 (0.70-0.74)	0.75 (0.73-0.77)
Mixed or multiple ethnic groups	1.01 (0.71-1.45)	1.01 (0.71-1.45)	0.76 (0.68-0.84)	0.79 (0.71-0.87)	0.80 (0.75-0.85)	0.80 (0.75-0.85)	0.83 (0.78-0.87)	0.84 (0.80-0.89)
Pakistani	1.27 (1.06-1.51)	1.12 (0.94-1.33)	2.42 (2.34-2.51)	2.09 (2.02-2.16)	0.93 (0.89-0.96)	0.86 (0.83-0.90)	0.67 (0.65-0.69)	0.63 (0.61-0.65)
Indian	3.19 (2.75-3.71)	2.96 (2.55-3.44)	1.42 (1.34-1.52)	1.34 (1.26-1.43)	0.74 (0.70-0.78)	0.72 (0.68-0.76)	0.79 (0.76-0.83)	0.78 (0.75-0.82)
Bangladeshi	1.69 (0.94-3.06)	1.75 (0.97-3.15)	1.59 (1.35-1.87)	1.93 (1.63-2.27)	0.79 (0.68-0.91)	0.82 (0.71-0.95)	0.64 (0.56-0.74)	0.69 (0.61-0.79)
Chinese	0.37 (0.24-0.58)	0.35 (0.22-0.55)	0.45 (0.40-0.50)	0.43 (0.38-0.49)	0.36 (0.33-0.39)	0.35 (0.32-0.38)	0.52 (0.49-0.55)	0.52 (0.49-0.55)
Other Asian	5.43 (4.71-6.25)	5.39 (4.68-6.21)	1.38 (1.27-1.49)	1.42 (1.31-1.53)	0.61 (0.57-0.66)	0.61 (0.57-0.66)	0.87 (0.83-0.92)	0.91 (0.86-0.95)

African	2.32 (1.88-2.88)	2.21 (1.79-2.74)	1.21 (1.12-1.30)	1.17 (1.08-1.27)	0.81 (0.76-0.86)	0.80 (0.75-0.85)	0.75 (0.71-0.79)	0.74 (0.70-0.78)
Caribbean	1.17 (0.66-2.05)	1.16 (0.66-2.05)	0.85 (0.70-1.02)	0.87 (0.72-1.05)	0.78 (0.69-0.88)	0.77 (0.68-0.87)	0.93 (0.85-1.03)	0.94 (0.85-1.03)
Arab	1.16 (0.70-1.93)	1.09 (0.66-1.81)	1.22 (1.07-1.39)	1.18 (1.04-1.35)	0.78 (0.71-0.87)	0.76 (0.69-0.84)	0.61 (0.55-0.67)	0.60 (0.55-0.66)
Other Ethnic Group	1.16 (0.64-2.10)	1.12 (0.62-2.02)	1.13 (0.95-1.34)	1.11 (0.93-1.32)	0.75 (0.66-0.86)	0.74 (0.64-0.84)	0.71 (0.63-0.80)	0.71 (0.64-0.80)

HR (95%CI) = Hazard ratio (95% Confidence Interval)- wave 1: March 1, 2020 to July 31, 2020- wave 2: August 1, 2020 to April 30, 2021- wave 3: May 1, 2021 to December 17, 2021- wave 4 : December 18, 2021 to April 17, 2022
